# RBP2 stabilizes slow Cav1.3 Ca^2+^ channel inactivation properties of cochlear inner hair cells

**DOI:** 10.1007/s00424-019-02338-4

**Published:** 2019-12-17

**Authors:** Nadine J. Ortner, Alexandra Pinggera, Nadja T. Hofer, Anita Siller, Niels Brandt, Andrea Raffeiner, Kristina Vilusic, Isabelle Lang, Kerstin Blum, Gerald J. Obermair, Eduard Stefan, Jutta Engel, Jörg Striessnig

**Affiliations:** 1 Department of Pharmacology and Toxicology, Institute of Pharmacy, Center for Molecular Biosciences Innsbruck, University of Innsbruck, Innsbruck, Austria; 2 Department of Biophysics and CIPMM, Saarland University, Homburg, Germany; 3 Institute of Biochemistry, Center for Molecular Biosciences Innsbruck, University of Innsbruck, Innsbruck, Austria; 4 Division of Physiology, Medical University Innsbruck, Innsbruck, Austria; 5 Division Physiology, Karl Landsteiner University of Health Sciences, Krems, Austria

**Keywords:** RIM-binding protein, Cav1.3, L-type Ca^2+^ channel, Inner hair cells, Ribbon synapse, Inactivation

## Abstract

Cav1.3 L-type Ca^2+^ channels (LTCCs) in cochlear inner hair cells (IHCs) are essential for hearing as they convert sound-induced graded receptor potentials into tonic postsynaptic glutamate release. To enable fast and indefatigable presynaptic Ca^2+^ signaling, IHC Cav1.3 channels exhibit a negative activation voltage range and uniquely slow inactivation kinetics. Interaction with CaM-like Ca^2+^-binding proteins inhibits Ca^2+^-dependent inactivation, while the mechanisms underlying slow voltage-dependent inactivation (VDI) are not completely understood. Here we studied if the complex formation of Cav1.3 LTCCs with the presynaptic active zone proteins RIM2α and RIM-binding protein 2 (RBP2) can stabilize slow VDI. We detected both RIM2α and RBP isoforms in adult mouse IHCs, where they co-localized with Cav1.3 and synaptic ribbons. Using whole-cell patch-clamp recordings (tsA-201 cells), we assessed their effect on the VDI of the C-terminal full-length Cav1.3 (Cav1.3_L_) and a short splice variant (Cav1.3_42A_) that lacks the C-terminal RBP2 interaction site. When co-expressed with the auxiliary β3 subunit, RIM2α alone (Cav1.3_42A_) or RIM2α/RBP2 (Cav1.3_L_) reduced Cav1.3 VDI to a similar extent as observed in IHCs. Membrane-anchored β2 variants (β2a, β2e) that inhibit inactivation on their own allowed no further modulation of inactivation kinetics by RIM2α/RBP2. Moreover, association with RIM2α and/or RBP2 consolidated the negative Cav1.3 voltage operating range by shifting the channel’s activation threshold toward more hyperpolarized potentials. Taken together, the association with “slow” β subunits (β2a, β2e) or presynaptic scaffolding proteins such as RIM2α and RBP2 stabilizes physiological gating properties of IHC Cav1.3 LTCCs in a splice variant-dependent manner ensuring proper IHC function.

**Electronic supplementary material:**

The online version of this article (10.1007/s00424-019-02338-4) contains supplementary material, which is available to authorized users.

## Introduction

Cav1.3 channels, which belong to the L-type family of voltage-gated Ca^2+^ channels [1, 70], confer > 90% of Ca^2+^ currents in cochlear inner hair cells (IHCs) and are essential for hearing [5, 51]. In IHCs, Cav1.3 channels are localized at the presynaptic active zone [11, 13, 64]. Their unique biophysical properties allow fast and sustained Ca^2+^ signals, making them ideally suited to couple the graded sound-evoked IHC receptor potential to Ca^2+^-dependent glutamate release. These properties include a fast activation time course, activation at hyperpolarized potentials, and very slow inactivation kinetics [20, 24, 31, 32, 34, 44, 51, 66]. Whereas fast activation and a negative activation voltage range are typical hallmarks of Cav1.3 channels in many electrically excitable cells [38, 39, 44, 70], their particularly slow inactivation kinetics are unique to IHCs. Two fundamental mechanisms drive inactivation of voltage-gated Ca^2+^ channels (VGCCs): Ca^2+^- (CDI) and voltage- (VDI) dependent inactivation [3, 22, 70, 73]. Fast CDI of Cav1.3 is mediated by Ca^2+^-induced conformational changes of calmodulin (CaM), which binds to N- and C-terminal motifs of the pore-forming α1 subunit [10]. In the absence of CDI, VDI is the predominant inactivation mechanism and can be measured using permeating ions unable to activate CaM, such as Ba^2+^ [6, 10]. VDI is likely induced by a “hinged-lid” mechanism involving residues at the intracellular activation gate (formed by S6 helices) [59, 61] as well as conformational changes within the selectivity filter and the pore-lining S6 segments (like in voltage-gated Na^+^ channels [45]). However, in IHCs, both CDI and VDI are extremely slow. Suppression of CDI is mediated by CaM-like Ca^2+^-binding proteins, such as CaBP2, which are abundantly expressed in IHCs [48, 55, 69] and inhibit CaM-mediated CDI [15, 68]. In contrast, the molecular basis for the uniquely slow VDI in IHCs is incompletely understood, although several factors are known to modulate VDI. For example, accessory β subunits, required for proper gating of VGCCs [70] including Cav1.3 [31], can modify channel kinetics. Indeed, certain splice variants of β2 subunits, the major isoform expressed in IHCs, are anchored to the lipid bilayer through their palmitoylated (β2a) or positively charged (β2e) N terminus and can slow VDI [18, 27, 52]. However, it is currently unclear which β2 splice variants are present in IHCs. Although β2 deficiency reduces current densities by about 70%, VDI remains unaffected in residual Cav1.3 currents [42]. This points toward other mechanisms that stabilize slow VDI also of Cav1.3 channels associated with other β subunits, such as β3 [42], which has been consistently detected in IHCs by PCR [33, 42] and transcriptome analysis [35, 37]. Also alternative splicing of Cav1.3 α1 subunits has pronounced effects on Cav1.3 inactivation, but this mainly affects CDI as shown for C-terminal alternative splicing into long (Cav1.3_L_) and several short variants, without a major impact on VDI [57, 62, 70]. Finally, similar to reduced CDI, slow VDI might also be mediated by Cav1.3 protein interactions unique to IHCs. Presynaptic VGCC abundance and clustering at release sites is tightly regulated by proteins of the presynaptic active zone such as bassoon, RIM2α, RIM2β, and RIM-binding proteins (RBPs) [17, 23, 32]. Cav1.3 channels are closely positioned next to readily releasable synaptic vesicles at the IHC ribbon synapse in a similar fashion, and it is therefore possible that interaction with presynaptic proteins also modulates Cav1.3 kinetics. This hypothesis is supported by our previous observation that RIM2α binding to the β3 subunit stabilizes slow Cav1.3 VDI but not to the extent as observed in IHCs [18]. However, in that study, we have only studied the effect of RIM2α on Cav1.3_L_ but not on C-terminally short splice variants, which are also expressed in IHCs [53].

**Fig. 1:**
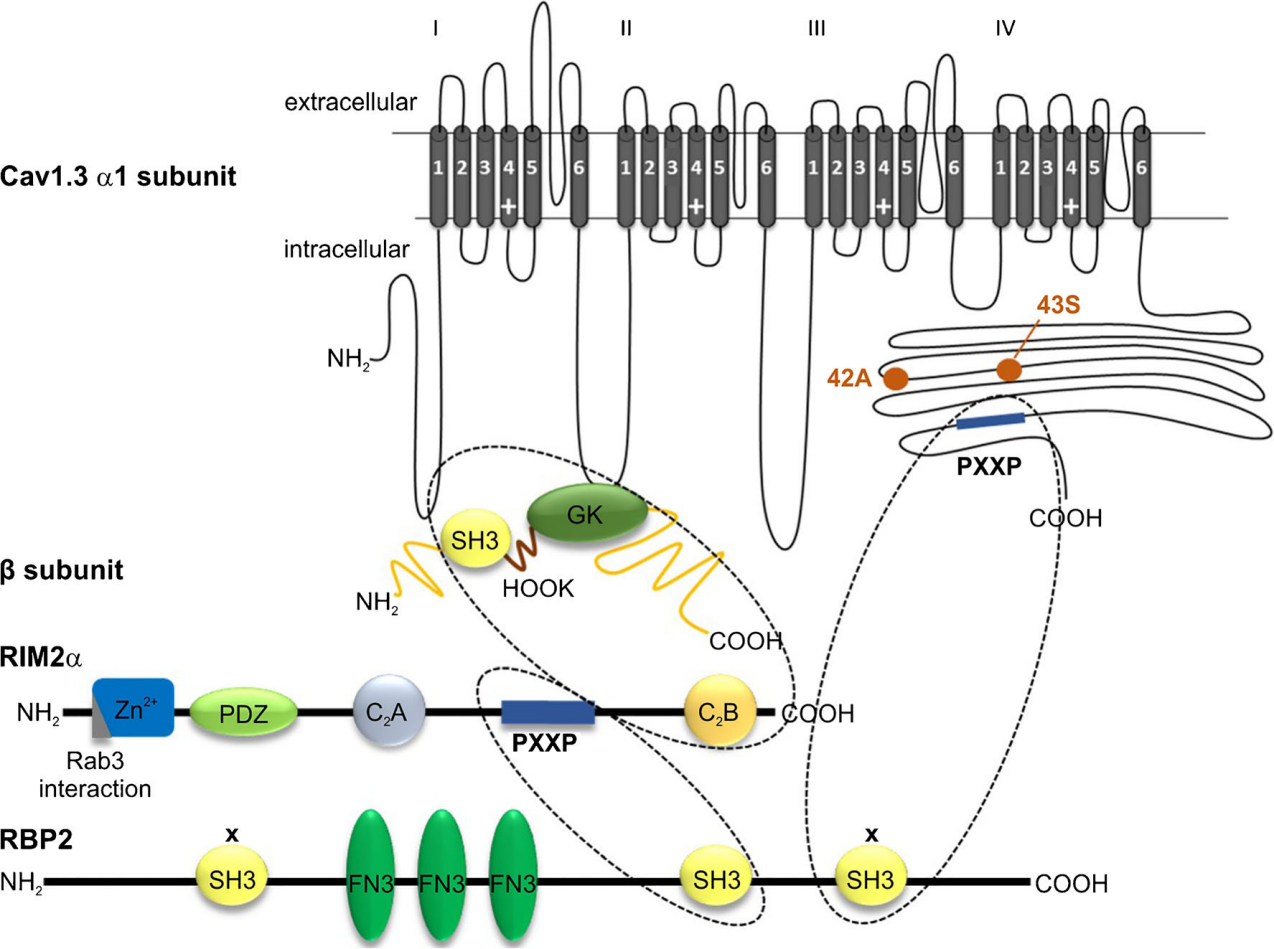
Interaction of RBP2 with Cav1.3 channels. RIMs and RBPs are multidomain proteins [41, 46]. All RIM isoforms (RIM1α and 1β; RIM2α, 2β, and 2γ; RIM3γ and RIM4γ) bind via their C_2_B domain to the auxiliary β subunit of the Ca^2+^ channel complex. Disruption of the SH3 or GK domain in the β subunit prevents the interaction with RIM [28]. All three RBP isoforms contain three SH3 domains and two (RBP3) or three (RBP1 and 2) FN3 domains [41]. The second SH3 domain of RBP binds to the proline-rich region (PXXP) present only in RIMα or β isoforms, located between the two C_2_ domains. The other SH3 domains, marked by “x,” in turn can interact with a proline-rich region (PXXP) localized in the full-length Cav1.3 C terminus [23]. Note that incorporation of alternative exons 42A and 43S leads to short C-terminal splice variants (Cav1.3_42A_ or Cav1.3_43S_, respectively; C-terminal ends indicated by orange dots) lacking the PXXP interaction site. AID, α-interaction domain; FN3, fibronectin 3 domain; GK, guanylate-kinase like domain; PXXP, proline-rich region; SH3, SRC homology 3 domain; Zn^2+^, zinc finger domain. Note that RIM may also interact via its C_2_B domain with the C terminus of Cav1.3, but the interaction site is unknown [49]

Here we investigated the possibility that complex formation of Cav1.3 with RIM2α together with RBPs can explain slow VDI in IHCs. This hypothesis is particularly attractive because RBPs can bind through distinct SH3 domains simultaneously to β subunit-associated RIM and to the C terminus of long Cav1.3 splice variants (Fig. 1). This should result in a decrease in the conformational flexibility of the C terminus, a mechanism known to stabilize slow VDI [30]. By co-expression of these large complexes in tsA-201 cells, we indeed found that RBP2 together with RIM2α slows VDI of Cav1.3_L_/β3 to a similar extent as in IHCs. This effect required the presence of the long C terminus, which contains the RBP2 interaction site. In contrast, the presence of RIM2α alone was sufficient to slow inactivation of the C-terminally short Cav1.3_42A_ channel variant. When β2a or β2e subunits, which already strongly reduce VDI, were part of the channel complex, neither RIM2α nor RBP2 were required for slow inactivation or could further modulate it. In summary, we show that IHC-like slow VDI is controlled by scaffolding proteins of the presynaptic active zone as well as by β2 subunit splice variants in a Cav1.3 splice variant-dependent manner.

## Material and methods

### Animals

All experiments were carried out in accordance with the European Communities Council Directive (86/609/EEC) and approved by the regional board for scientific animal experiments of the Saarland University, Germany. Additional ethics approval was not required according to the local and national guidelines. NMRI mice were purchased from Charles River (Sulzfeld, Germany) and were housed with free access to food and water at an average temperature of 22 °C and a 12-h light–dark cycle. Mice of either sex were used. Cochleae were dissected from temporal bones after mice had been sacrificed by cervical dislocation under isoflurane anesthesia at ages ≥P10 or by decapitation (P5–P6).

### RNA isolation, reverse transcription, and qualitative PCR analysis (RT-PCR)

PCR was performed on cDNA synthesized from IHCs, which were selectively collected with glass pipettes with a tip diameter of 7–10 μm under micromanipulator and microscope control as described [33, 53] from P5–31 NMRI mice. Care was taken to prevent cross-contamination between OHCs and IHCs. For reverse transcription (RT), 40–130 individually collected IHCs and 110–600 OHCs [53] were lysed by three freezing and thawing cycles in liquid nitrogen; 9 μl of cell lysate was mixed with 5 μM random primers pd.(N)_6_ (Thermo Fisher Scientific, Waltham, MA, USA) and 0.5 mM dNTPs (NEB, Ipswich, MA, USA) to a final volume of 50 μl, incubated for 5 min at 65 °C and transferred back on ice for 1 min. An RT mix (8 μl; Thermo Fisher Scientific, Waltham, MA, USA) consisting of 5× reverse transcription buffer, 10 mM dithiothreitol, 2 U/μl RNaseOUT recombinant ribonuclease inhibitor, and 10 U/μl SuperScript III reverse transcriptase was added to reach a final volume of 20 μl. For the cDNA first strand synthesis, the final reaction mix was first incubated at 25 °C for 5 min, 50 °C for 150 min followed by 70 °C for 30 min. The cDNA was stored at − 20 °C until PCR analysis.

For RNA extraction of whole brains or whole cochlea, mice were sacrificed by cervical dislocation under isoflurane anesthesia and decapitated. Whole brains were quickly removed without cerebellum, snap frozen in liquid nitrogen, and stored at − 80 °C. For cochlea preparations, the bony cochlear capsule was carefully removed and the extracted cochlear helix was immediately snap frozen in liquid nitrogen. Total RNA was purified using Qiagen RNeasy lipid tissue mini kit (Qiagen, GmbH, Hilden, Germany). Samples were lysed using 6 ml (whole brain) or 1 ml (whole cochlea) of phenol/guanidine-based QIAzol lysis reagent (Qiagen, GmbH, Hilden, Germany). The tissue was disrupted, homogenized, and processed according to the manufacturer’s protocol. An optional on-column DNase digestion was performed to reduce genomic DNA contamination. For the final elution, two times 30 μl nuclease-free water was used. The RNA concentration was determined photometrically yielding approximately 1–2 μg/μl RNA with high purity; 1 μg or 13 μl of total RNA was reverse transcribed using Maxima H Minus First Strand cDNA Synthesis kit with random hexamer primers (Thermo Fisher Scientific, Waltham, MA, USA).

For detection of N-terminal β2 splice variants, RIM and RBP isoforms in cochlear IHCs, nested PCR was used. Initial denaturation was performed at 94 °C for 3 min, followed by 30 (first PCR) or 25 (second PCR) cycles of 30 s denaturation at 94 °C, annealing for 30 s at variable temperatures depending on the melting temperature of the primers followed by an extension step at 72 °C. The extension time was determined based on the size of the amplified DNA segment and was calculated according to the manufacturer’s instructions (for Taq polymerase 1 min/kb). A final extension step was performed at 72 °C for 7 min. The reaction mix for the first PCR standardly contained 1–2 μl cDNA (corresponding to 50–100 ng RNA equivalent), 2 μl 10× BioTherm Taq buffer containing 15 mM MgCl_2_ (GeneCraft, Cologne, Germany), 2 μl 2 mM dNTPs (Thermo Fisher Scientific Inc., Waltham, MA, USA), 1 μl DMSO (Merck Millipore, Burlington, MA, USA), 1 μl 10 μM outer forward and outer reverse primer, respectively, 0.25 μl BioTherm Taq polymerase (GeneCraft, Cologne, Germany), and nuclease-free water (Thermo Fisher Scientific Inc., Waltham, MA, USA) to a total volume of 20 μl. The second PCR was performed with the same reaction mix, except that 0.2–1 μl of the first reaction was used as template together with 1 μl 10 μM inner forward and inner reverse primers, respectively. IHC and OHC preparations were used as a template. Whole cochlea or whole brain preparations served as positive controls. Samples without template served as negative controls. All samples were supplemented with 1 ng/μl of poly-dC-DNA (Midland Certified Reagent Company Inc., Midland, TX, USA) in order to reduce self-aggregation of DNA or adhesion to sticky tubes. Primers were purchased from Eurofins (Eurofins MWG Operon, Ebersberg, Germany) and designed to recognize all N-terminal β2 (β2gen) splice variants as well as specifically β2a, β2b, β2c, β2d, and β2e splice variants. Different splice variant-specific or unspecific forward primers were combined with several reverse primers as given in Table S1. The identity of all detected transcripts was confirmed by sequencing.

Primers for detection of RIM and RBP isoforms were purchased from Biomers. Specific primers for mouse RIM isoforms were taken from [18] and are given in Table S1. Specific primers for mouse RBP isoforms are given in Table S1. They were designed based on the indicated accession numbers. Specificity was verified by basic local alignment search tool (BLAST, http://blast.ncbi.nlm.nih.gov/Blast.cgi). For RBP1, outer primers were located in exons 7 and 13, and for RBP2, in exon 10 and exon 11. Primers for the detection of C-terminal long and short Cav1.3 splice variants were taken from [53] and are given in Table S1. The primers were within exon 42 and exon 45 and allowed to distinguish between the long and short splice variants containing long exon 43L or short exon 43S.

### Quantitative real-time PCR

The expression of β1–4 isoforms and N-terminal β2 splice variants was assessed using a standard curve method based on PCR fragments of known concentration [54]. Flanking primer pairs (Eurofins MWG Operon, Ebersberg, Germany) were designed to amplify templates for β1–4 standard curves using mouse whole brain cDNA (Table S2). PCR products were separated on 1.5% low melting point agarose gels (Agarose II, Cat# 0815, Amresco®, VWR, Radnor, PA, USA). Bands were excised and DNA was extracted using Nucleospin Extract II columns (Macherey-Nagel, Düren, Germany). Fragments were sequenced (Eurofins MWG Operon, Ebersberg, Germany) to confirm the integrity of the obtained fragments. N-terminal β2 splice variant fragments were amplified from mouse whole brain cDNA using primers spanning the 5′ UTR and exon 5 (Table S2). The fragments were subsequently cloned into the pGFPminus vector [4, 31]. In order to generate DNA templates of known concentrations for quantitative real-time PCR (qRT-PCR) standard curves, the concentration of the amplified (β1–β4) or digested fragments (β2a–β2e) was determined using the Quant-iT™ PicoGreen™ dsDNA Assay Kit (Cat# P11496; Thermo Fisher Scientific, Waltham, MA, USA). Subsequently, standard curves were generated using a serial dilution ranging from 10^7^ to 10^1^ DNA molecules in water containing 1 μg/ml of poly-dC-DNA (Midland Certified Reagent Company Inc., Midland, TX, USA). qRT-PCRs of standard curves and samples were performed as described previously [54]. For standard curve properties and limit of detection (LOD) and limit of quantification (LOQ), see Table S3. The samples containing the following were mixed standardly: the respective TaqMan gene expression assay (see Table S2; Thermo Fisher Scientific, Waltham, MA, USA), TaqMan Universal PCR Master Mix for β1–4 isoforms (Thermo Fischer Scientific, Waltham, MA, USA), or Luna® Universal Probe qPCR Master Mix for β2a–β2e splice variants (New England Biolabs GmbH, Ipswich, MA, USA) and poly-dC-water (1 μg/ml). Samples for qRT-PCR quantification (50 cycles) of β1–4 isoforms contained cDNA from 4 to 10 IHCs (P6 or P20), 12–24 OHCs (P6 or P20), and 2 μl organ of Corti preparations (P5) from one (IHC) or two (OHC, organ of Corti) independent preparations. Samples for qRT-PCR quantification of β2a–β2e splice variants contained cDNA from 1.75 to 13 IHCs (P22), 12 OHCs (P24), or 5 ng of total RNA equivalent of cDNA (cochlea; P23) from three independent RNA preparations of three NMRI mice. Specificity of the custom-designed assays recognizing β2a, β2b, β2c+d (both variants detected with the same assay), and β2e was confirmed using different DNA ratios of corresponding and mismatched β2 splice variant fragments (data not shown). Importantly, all four assays recognized only the corresponding fragment (low CT value; ≤ 22) even in the presence of a 10-fold higher concentration of the mismatching DNA fragments. Samples without template served as negative controls. As independent quality control, the expression of the endogenous control gene hypoxanthine phosphoribosyl-transferase 1 (Hprt1; Mm00446968_m1) was routinely measured [12, 58]. Molecule numbers were calculated for each assay based on their respective standard curve. Analyses were performed using the 7500 Fast System (Thermo Fisher Scientific, Waltham, MA, USA).

### Antibodies

*Immunohistochemistry*: anti-RBP2 (guinea pig polyclonal, 1:1000; kind gift of Eckart Gundelfinger; specifically recognizes RBP2 as verified by the knockout, [21]); anti-Cav1.3 (rabbit polyclonal, 1:500; Alomone Labs; [53]); anti-CtBP2/RIBEYE (mouse monoclonal, 1:200; BD Biosciences); Alexa488 anti-guinea pig (goat polyclonal; 1:500; Invitrogen), Alexa568 anti-mouse (goat polyclonal; 1.500; Invitrogen), Cy3 anti-rabbit (donkey polyclonal; 1:1500; Jackson Immuno Research); *co-immunoprecipitation*: affinity purified anti-RBP2-1318 (rabbit polyclonal; 1:100; directed against amino acids 261–284, NCBI reference sequence NP_001074857.1); anti-GFP (mouse monoclonal; 4 μl of 0.4 μg/μl; Roche); peroxidase-conjugated secondary antibodies: anti-rabbit (goat polyclonal; 1:20,000; Sigma-Aldrich); anti-mouse (goat polyclonal; 1:5000; Sigma-Aldrich); *GST (glutathione-S-transferase) pull-down*: anti-GAPDH (rabbit monoclonal; 1:1000; Cell Signaling Technology); anti-HA (mouse monoclonal κ16B12; 1:1000; Biozym); affinity-purified anti-Cav1.3α1_2022–2138_ (rabbit polyclonal; 1:1000; [51]). Peroxidase-conjugated secondary antibodies: anti-mouse (polyclonal goat; 1:3000; Roth) and anti-rabbit (polyclonal goat; 1:3000; Roth).

### Immunohistochemistry

Immunolabeling was performed on whole-mount organs of Corti of 4-week-old NMRI mice as described [12] using Zamboni’s fixative for 8 min on ice. Specimens were double-labeled by simultaneous incubation with an antibody against RBP2 and antibodies directed against Cav1.3 or CtBP2/RIBEYE, embedded with Vectashield mounting medium with DAPI (Vector, UK) and viewed using a confocal Zeiss LSM 710 (Zeiss Microscopy GmbH, Göttingen, Germany) with a ×63/1.4 NA oil objective.

### cDNA constructs

Primers for cloning of cDNA constructs are given in Table S4. GFP-tagged rat RIM2α is based on rRIM2α-pCMV5 (kindly provided by Susanne Schoch, AF199323.1, [63]) and was cloned into the pGFP^+^ vector to allow visual detection of protein expression as described previously [18].

The rat RBP2 constructs (RFP-rRBP2, HA-rRBP2) are based on rRBP2-pEGFP-C1 (GFP tag on the N terminus; kindly provided by Susanne Schoch (NM_001100488.2)). To generate the HA-rRBP2 construct, the HA tag was introduced via SOE-PCR on the N terminus of the RBP2 and rRBP2-pEGFP-C1 was used as template. The resulting PCR fragment was subcloned into pJET1.2/blunt (CloneJet PCR cloning Kit # K1232 Fermentas) according to the blunt end protocol of the manufacturer (pJET1.2_PCR_fragment). In order to obtain full-length RBP2, rRBP2-pEGFP-C1 was digested with XmaI and the resulting fragment was cloned into pJET1.2_PCR_fragment. The correct direction of the insertion was confirmed by restriction digestion. Full-length HA-RBP2 was then cloned back into the pEGFP-C1 vector after removal of the GFP-tag (template rRBP2-pEGFP-C1, restriction sites NdeI and SalI). To generate the RFP-rRBP2, rRBP2 was cloned form rRBP2-pEGFP-C1 using MfeI and XhoI sites into the pmRFP-C1 vector [18] which was digested with MfeI and SalI.

GST-Cav1.3 42_C-term_ (aa 1449–2137), GST-Cav1.3 42A_C-term_ (aa 1449–1632), and GST-Cav1.3 43S_C-term_ (aa 1449–1679) were cloned into GST-pGEX 5x-1 via BamHI and XhoI sites which were introduced into the C termini through the respective forward and reverse primers. GST-Cav1.3 42_C-term_ was cloned from YFP-Cav1.3 42_C-term_. GST-Cav1.3 42A_C-term_ and GST Cav1.3 43S_C-term_ were cloned from C-terminally short Cav1.3 α1 subunits Cav1.3_42A_ and Cav1.3_43S_ as described [4, 57].

The cloning of GST-RIIβ in pGEX-4T-2 [2], GST-maxP14 in pET42a [14], and YFP-Cav1.3_C-term_ (Cav1.3-EF-preIQ-IQ-PostIQ-aa1453–2137, full-length C terminus; [57]) has been described previously.

For cDNA constructs for electrophysiology, human full-length (Cav1.3_L_, GenBank accession number EU363339) and C-terminally short Cav1.3 α1 subunits (Cav1.3_42A_) are described in detail in [4, 57] and [50]. The following LTCC auxiliary subunits were used: β3 (rat, NM_012828), β2a (rat, M80545), β2e (β2aN5, FM872407, a kind gift of V. Flockerzi, Saarland University, Homburg, [36]), and α2δ1 (rabbit, NM_001082276).

### Cell culture of HEK293 and tsA-201 cells, transfection, and preparation of cell lysates

HEK293 cells (GST pull-down) or HEK293 cells stably expressing an SV40 temperature-sensitive T-antigen (tsA-201 cells, ECACC: 96121229; electrophysiology and co-immunoprecipitation) were cultured in Dulbecco’s modified Eagle’s medium (DMEM; Sigma-Aldrich, D6546) supplemented with 10% FBS (Gibco, 10270-106), 2 mM l-glutamine (Gibco, 25030-032), penicillin (10 U/ml; Sigma-Aldrich, P-3032), and streptomycin (10 μg/ml; Sigma-Aldrich, S-6501). Cells were maintained at 37 °C and 5% CO_2_ and split at ~ 80% confluency using 0.05% trypsin for cell dissociation. The passage number did not exceed 20 passages.

For whole-cell patch-clamp experiments, tsA-201 cells were transfected with 3 μg of α1, 2 μg of β, 2.5 μg of α2δ1, and 3 μg of GFP-rRIM2α and/or RFP-RBP2 using the Ca^2+^-phosphate precipitation method as previously described [44]. Cells were then plated onto a 35-mm culture dish precoated with poly-l-lysine, kept at 30 °C and 5% CO_2_, and subjected to electrophysiological measurements 48–72 h after transfection. When no GFP- or RFP-labeled construct was used, eGFP was co-transfected to visualize transfected cells.

For GST pull-down and co-immunoprecipitation experiments, cells were transfected with cDNA constructs of interest using TransFectin Lipid Reagent (BioRad; 170-3352) with 2 μg DNA per 100 mm dish (DNA (μg) to lipid (μl) ratio of 1:2). For the preparation of whole-cell lysates, growth medium was removed and cells were suspended in 1× PBS. After centrifugation at 650×*g* for 2 min at room temperature, the cell pellet was washed twice with PBS and resuspended in ice-cold lysis buffer (for GST pull-down: 1× PBS, 0.5% (v/v) Triton X-100; protease inhibitors: 1 μg/ml aprotinin, 1 μg/ml leupeptin, 1 μg/ml pepstatin A, 100 μM sodium orthovanadate, 100 μM sodium pyrophosphate, 500 μM sodium fluoride; for co-immunoprecipitation: 1× PBS, 0.5% Triton X-100; protease inhibitors: 1 μg/ml aprotinin, 1 μg/ml leupeptin, 1 μg/ml pepstatin, 10 μg/ml trypsin inhibitor, 0.5 mM benzamidine, 0.2 mM phenylmethylsulfonylfluoride, 2 mM iodacetamide), sheared 10 times with a needle, and kept on ice for 10–15 min. The lysate was cleared by centrifugation for 45–60 min at 20,000×*g* at 4 °C.

### GST pull-down

For the expression and purification of recombinant proteins, GST-fusion proteins were expressed in *Escherichia coli* Rosetta(DE3)pLysS grown at 37 °C to an optical density of 0.5 at 600 nm. Recombinant protein synthesis was induced for 4 h at 30 °C by the addition of isopropyl-β-d-thiogalactoside (IPTG) to a final concentration of 0.5 mM (pGEX) or 1 mM (pET30a). Bacteria were centrifuged at 6000×*g* for 15 min at 4 °C and resuspended in 8 ml GST bacteria lysis buffer (25 mM Tris-HCl pH 8.0, 150 mM NaCl). After adding 6 μl 10 mg/ml DNAseI and 8 μl 1 M MgCl_2_, bacteria were kept on ice and lysed three times at 90 bar (1.260 psi) using a French press. Recombinant fusion proteins were purified using Sepharose Glutathione 4B beads (GE Healthcare, 17-0756-01) suspended in GST buffer (25 mM Tris-HCl pH 8.0, 150 mM NaCl, 5% glycerol, 0.5% Triton X-100) and centrifuged at 2000×*g* for 3 min at 4 °C to collect the beads. Bacteria lysates were incubated with beads for 2 h at 4 °C using an overhead shaker. Beads were collected by centrifugation at 2000×*g* for 3 min at 4 °C and washed four times in GST buffer (2000×*g*, 3 min, 4 °C). After the last wash, the beads were resuspended in 300 μl GST buffer and stored at − 80 °C. To monitor the purity of the isolated proteins, the suspended beads were analyzed by SDS-PAGE and Coomassie Brilliant Blue staining. For pull-down experiments, GST-fusion proteins on glutathione Sepharose beads were incubated with cell lysates (see above) for 3 h at 4 °C using an overhead shaker. After binding, beads were washed four times with GST-lysis buffer as described above. Proteins were denatured by adding Laemmli buffer and subjected to SDS-PAGE and immunoblotting experiments [53].

### Co-immunoprecipitation experiments

The cell lysate (see above) was transferred to a fresh tube, and 0.4 mg/ml anti-GFP antibody or mouse IgG (reagent grade, Sigma-Aldrich I5381) together with 10 μl Protein G-sepharose 4B beads (Thermo Fisher, 101242) was added and incubated for 4 h at 4 °C on an overhead shaker. Samples were washed four times using washing buffer (1× PBS, 0.5% Triton X-100, 4 °C) and centrifuged at 13,000×*g* for 1 min. Proteins were denatured by adding Laemmli buffer and subjected to SDS-PAGE and immunoblotting experiments.

### Whole-cell patch-clamp recordings in tsA-201 cells

Electrodes with a resistance of 1.8–3.5 MΩ were pulled from glass capillaries (borosilicate glass, 64-0792, Harvard Apparatus, USA) using a micropipette puller (Sutter Instruments) and fire-polished with a MF-830 microforge (Narishige, Japan). tsA-201 cells were recorded in the whole-cell patch-clamp configuration using an Axopatch 200B amplifier (Axon Instruments, Foster City, CA). Recordings were digitized (Digidata 1322A digitizer, Axon Instruments) at 40 or 50 kHz, low-pass filtered at 5 kHz, and subsequently analyzed using pClamp 10.2 software (Axon Instruments). Current leak subtraction was applied either online (P/4 subtraction; *I*_Ba_–*V* protocol) or offline (5 s inactivation and steady-state inactivation protocol). Bath solution (in mM): 15 BaCl_2_, 150 choline-Cl, 1 MgCl_2_, 10 HEPES, adjusted to pH 7.3 with CsOH; pipette internal solution (in mM): 135 CsCl, 10 Cs-EGTA, 1 MgCl_2_, 10 HEPES, 4 ATP-Na_2_ adjusted to pH 7.4 with CsOH. Recordings between 100 and 1000 pA were selected and all voltages were corrected for a liquid junction potential of − 9.2 mV.

Ba^2+^ current–voltage (*I*_Ba_–*V*) relationships were obtained by applying a 20-ms long square pulse to various test potentials (Δ5 mV) starting from a holding potential (HP) of − 89 mV. The resulting *I*–*V* curves were fitted to the equation, *I* = *G*_max_(*V* − *V*_rev_)/(1 + exp[−(*V* − *V*_0.5_)/*k*]), where *I* is the peak current amplitude, *G*_max_ is the maximum conductance, *V* is the test potential, *V*_rev_ is the extrapolated reversal potential, *V*_0.5_ is the voltage of half-maximal activation, and *k* is the slope factor. The voltage dependence of conductance (*G*) was fitted using the following Boltzmann relationship: *G* = *G*_max_/(1 + exp[−(*V* − *V*_0.5_)/*k*]). The amount of voltage-dependent inactivation (VDI) during a 5-s depolarizing pulse from a HP of − 89 mV to the *V*_max_ of each individual cell was quantified by calculating the residual Ba^2+^ current fraction after 250, 500, 1000, or 5000 ms (r250, r500, r1000, r5000). The voltage dependence of inactivation was examined using a 20-ms long test pulse from a HP of − 89 mV to *V*_max_ before and after holding the cell for 5 s at various conditioning test potentials (30 s intersweep interval to allow recovery from inactivation in between sweeps). Steady-state inactivation parameters were obtained by fitting the data to a modified Boltzmann equation, *G* = (1 − *G*_max_)/(1 + exp[(*V* − *V*_0.5,inact_)/*k*_inact_]) + *G*_max_, where *V*_0.5,inact_ is the voltage of half-maximal inactivation and *k*_inact_ is the inactivation slope factor.

### Statistics

Data analysis was performed using Clampfit 10.2 (Axon Instruments) and Sigma Plot 12.5 (Systat Software Inc.). Values are presented as mean ± standard error (SEM) for the indicated number of experiments (*n*). Statistical significance was determined by unpaired Student’s *t* test or one-way ANOVA (with Bonferroni’s post hoc test) as indicated using GraphPad Prism 5.1 software (GraphPad Software Inc.). Statistical significance was set at *p* < 0.05.

## Results

We have previously found that co-expression of RIM2α can inhibit VDI of Cav1.3 [18]. Due to their multidomain structure, RIM proteins are able to interact with a variety of other proteins (for review, see [46]). This also includes RIM-binding protein 2 (RBP2), which has been shown to bind to the Cav1.3_L_ C terminus [23]. The C-terminal tail of LTCCs has also been implicated in channel inactivation since restricting its flexibility by membrane anchoring could slow VDI [30]. We therefore hypothesized that RBP2 could establish an intramolecular cross-link by binding simultaneously to the Cav1.3 C terminus and to RIM2α bound to the auxiliary β subunit (Fig. 1) and thereby further inhibit VDI to an extent similar as observed in IHCs.

### Expression of RBPs in mouse cochlear IHCs

**Fig. 2:**
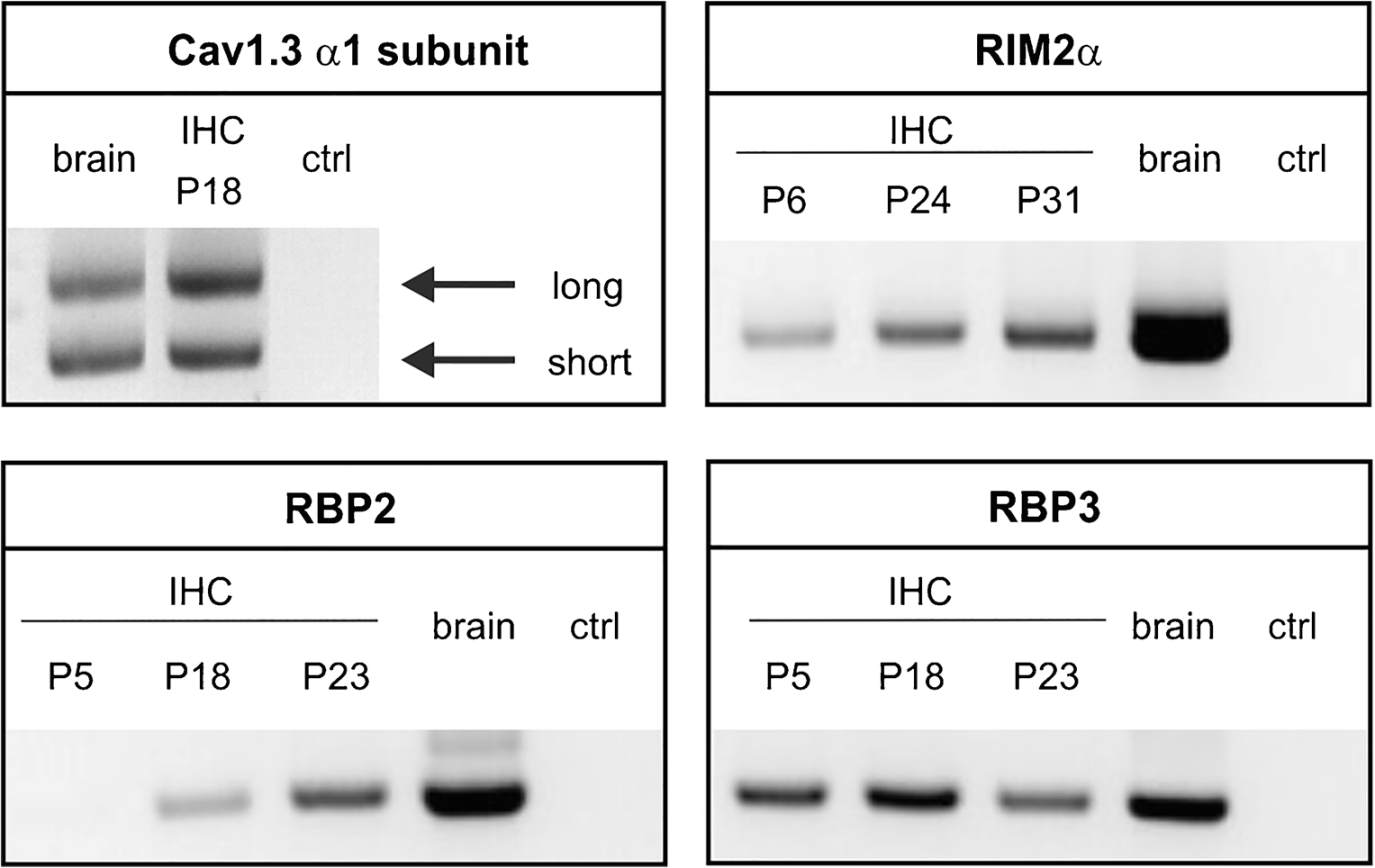
RIM, RBP, and Cav1.3 α1 subunit expression in IHCs. Control experiments in IHC preparations revealed the expected transcripts of long (containing exon 43) and short C-terminal splice variants (containing exon 43S) of Cav1.3 α1 subunits (top left). RIM2α was reliably detected in IHCs (4 out of 4 independent preparations) before (P6) and after hearing onset (after P12) (top right). RBP1 was the only isoform, which could not be detected in IHCs at any tested developmental stage (cDNA preparations from 5 different mice at different postnatal days, not shown). RBP2 transcripts (bottom left) were found only in 1 out of 5 different samples before hearing onset but were consistently detected in mature IHCs (8 out of 9 separate preparations). RBP3 transcripts (bottom right) were identified before as well as after hearing onset (6 out of 6 and 8 out of 10 independent samples, respectively). Brain samples from adult mice and reactions without template (“ctrl”) were used as positive and negative controls, respectively. Representative PCRs from > 3 independent experiments are shown

**Fig. 3:**
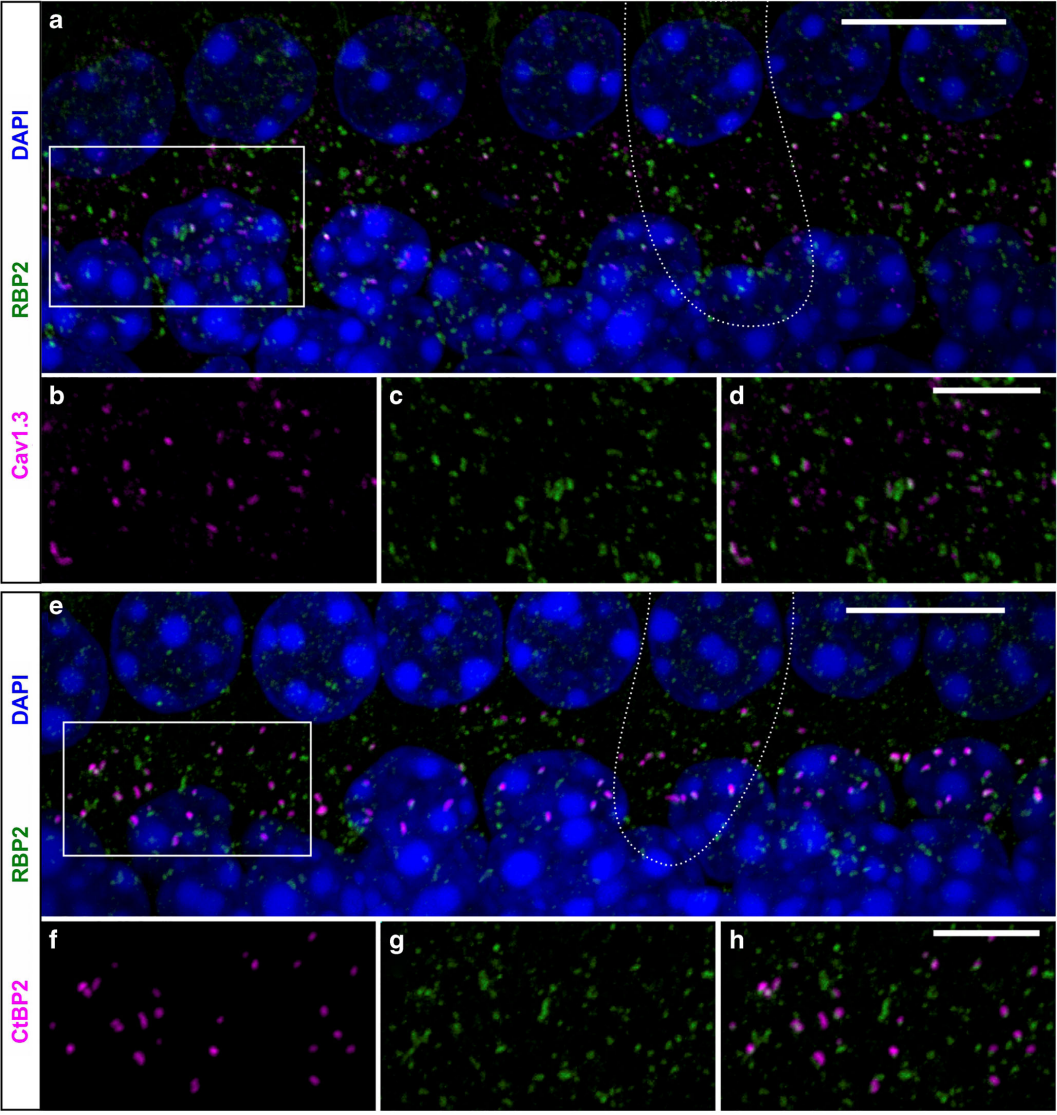
RBP2 co-localization with Cav1.3 at ribbon synapses in mouse IHCs. **a**–**h** Maximum intensity projection (MIP) of confocal stacks of whole-mount organs of Corti with stretches of 7–8 IHCs. **a**–**d** IHCs from the apical cochlear turn of a 4-week-old NMRI mouse co-immunolabeled for Cav1.3 and RBP2 demonstrate that almost every Cav1.3 cluster co-localized with RBP2 at the basolateral pole of the IHCs (**a**), which is shown in more detail in the enlargements of the box in **a** (**b**–**d**). **e**–**h** IHCs from the apical cochlear turn of a 4-week-old NMRI mouse co-immunolabeled for the ribbon synapse marker CtBP2 and RBP2 show that almost every ribbon co-localized with RBP2 at the basolateral pole (**e**), which is shown in more detail in the enlargements of the box in **e** (**f**–**h**). Nuclei stained in blue with DAPI are shown only in the merged images. The dotted lines in **a** and **e** outline the basolateral pole of one IHC in each specimen. Scale bars: **a**, **e**, 10 μm; **d**, **h**, 5 μm

We first determined which RIM and RBP isoforms are expressed in cochlear IHCs of apical turns at the indicated age using RT-PCR (Fig. 2). RIM2α transcripts were reliably detected with increasing abundance during postnatal development. RIM1α and RIM3 were only detected in brain samples as positive controls but not in IHCs (not shown). RBP1 was not found in IHCs (not shown), while RBP2 was consistently detected in adult IHCs after hearing onset (>P12), and similar to RIM2α, levels increased during postnatal development. In contrast, RBP3 transcripts were present throughout postnatal development. This was in line with a study showing an upregulation of brain RBP1 and RBP2 levels during brain development (P0–P15), while RBP3 levels were low and unchanged [41]. Since a recent transcriptome analysis [35] reported much higher levels of RBP2 compared to RBP3 in IHCs, high RBP3 expression has been found primarily outside the nervous system (mainly in mouse testis [41, 72]), and validated specific antibodies for mouse RBP3 are not available, we decided to focus on the RBP2 isoform and studied its localization in mouse IHCs. Indeed, Cav1.3 α1 subunits co-localized with RBP2 in IHCs as shown by double immunofluorescence labeling and confocal microscopy in IHCs of 4-week-old mice. Figure 3 illustrates that essentially all Cav1.3-positive clusters are also positive for RBP2 (Fig. 3a–d). In addition, RBP2 co-localized with the ribbon synapse marker CtBP2 (Fig. 3e–h) but was also detected in clusters outside ribbons, which is in agreement with its localization at presynaptic terminals of efferent olivocochlear neurons [32]. As we and others [18, 26] have shown, RIM2α co-localizes with synaptic ribbons and positively regulates the number of synaptic Cav1.3 channels [26]. In summary, our data demonstrate that Cav1.3 channels co-localize with both RIM2α and RBP2 at synaptic ribbons of IHCs. In contrast to a previous study [32], we can directly demonstrate the co-localization of RBP2 and Cav1.3 at the same ribbon.

### RBP2 interaction with long Cav1.3 C termini

**Fig. 4:**
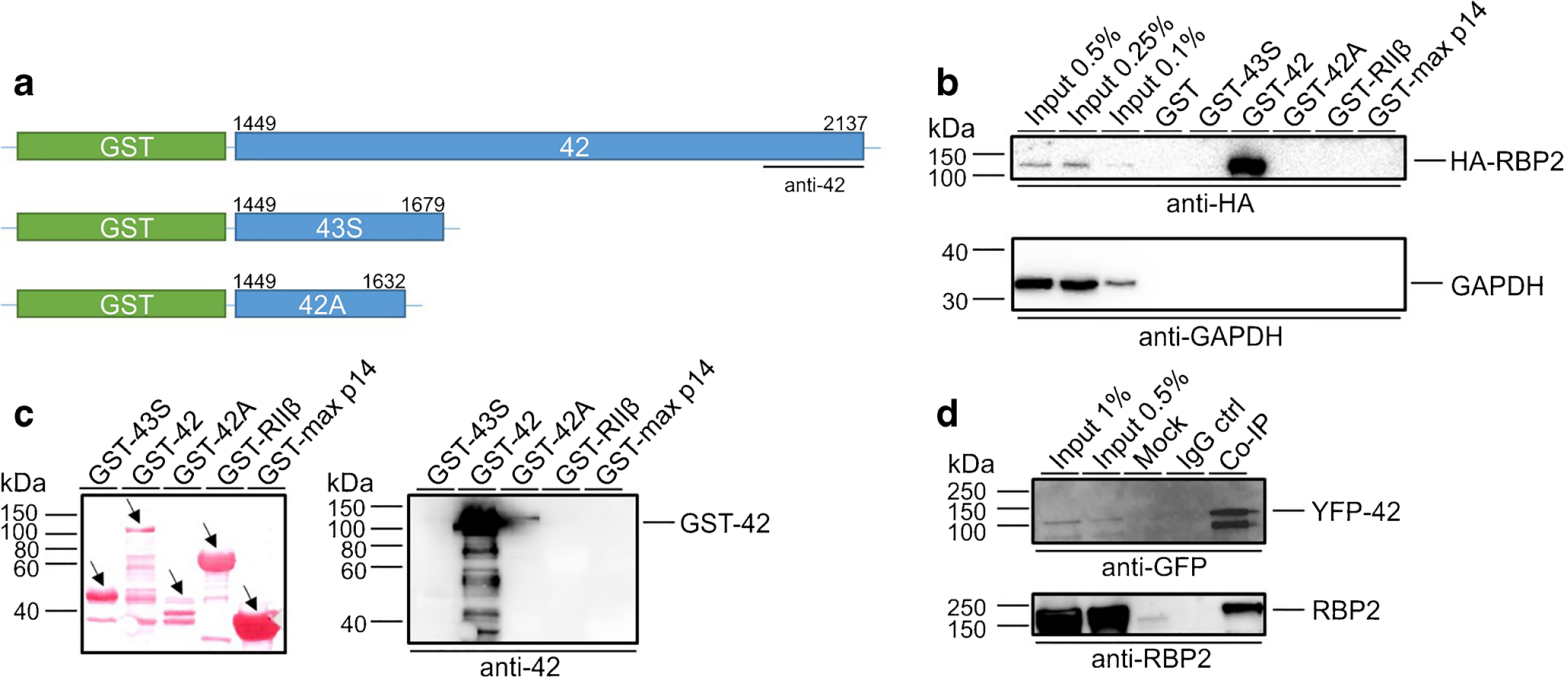
RBP2 interaction with Cav1.3 C-terminal splice variants. **a** Schematic representation of the Cav1.3 C-terminal GST-fusion proteins: GST-Cav1.3 42_C-term_ (GST-42), GST-Cav1.3 42A_C-term_ (GST-42A), and GST-Cav1.3 43S_C-term_ (GST-43S) including the binding position for the anti-Cav1.3α1_2022–2138_ antibody (anti-42) in the full-length C terminus. Numbers indicate the amino acid position in the Cav1.3 protein (GenBank™ accession number NM_000720). **b** GST pull-down of whole-cell extracts prepared from HEK293 cells transfected with HA-RBP2 with the indicated Cav1.3 C termini coupled to GST; 1 of 4 similar experiments is illustrated. Bound HA-RBP2 was visualized by western blotting using anti-HA. Anti-GAPDH staining served as a negative control. Input—0.5, 0.25, and 0.1% of the lysate. GST, GST-RIIβ, and GST-max p14 were control peptides not binding to HA-RBP2. Migration of molecular mass markers is indicated. **c** Left: Ponceau staining of GST-fusion proteins. Arrows indicate the migration of the full-length construct. Despite the partial degradation of GST-fusion proteins GST-42 and GST-42A, we observed selective protein–protein interactions between GST-42 and RBP2. Right: Immunoblot from panel **b** was stripped and the presence of GST-Cav1.3 42_C-term_ was verified by immunoblotting using anti-Cav1.3α1_2022–2138_ antibody directed against an epitope present only in the long C-terminal splice variant as illustrated in panel **a**. **d** Confirmation of HA-RBP2 interaction with the long Cav1.3 C terminus by co-immunoprecipitation of HA-rRBP2 expressed in tsA-201 cells with YFP-tagged long Cav1.3 C terminus (YFP-Cav1.3 42_C-term;_ YFP-42). Top: Verification of the presence of YFP-Cav1.3 42_C-term_ by immunoblotting using an YFP antibody. Bottom: Specific immunoprecipitation of RBP2 by Cav1.3 42_C-term_ (detection by anti-RBP2-1318). Input control—1 and 0.5% of the lysate. Mock: untransfected control

Since splicing within the Cav1.3 α1 C terminus, containing the proposed RBP2 interaction site, results in functionally distinct “long” (Cav1.3_L_) and “short” splice variants, we investigated splice variant-specific RBP2 interactions. Using in vivo labeling of long Cav1.3 α1 subunits, we have recently identified Cav1.3_L_ at IHC ribbon synapses [53]. However, transcripts for short Cav1.3 α1 subunit splice variants are also abundantly expressed in isolated IHCs (mostly resulting from alternative splicing of exons 42_A_ or 43_S_, Fig. 2, [53]) and are thus expected to contribute to Cav1.3 currents in IHCs. We therefore included one of the major short variants (Cav1.3_42A_) of Cav1.3 in our functional analysis to test the effect of the C terminus on the regulation by RIM2α and RBP2. This is of particular importance because RBP2 is predicted to bind to a proline-rich motif in the distal C terminus of Cav1.3_L_ that is absent in Cav1.3_42A_ (Fig. 1). To confirm the differential interaction of RBP2 with Cav1.3 C termini, we performed GST pull-down experiments with the long (GST Cav1.3 42_C-term_) and both major short C termini (GST-Cav1.3 42A_C-term_ and GST-Cav1.3 43S_C-term_) of Cav1.3 (for schematic representation see Fig. 4a). As depicted in Fig. 4b, only the long C terminus was able to specifically pull-down HA-tagged RBP2. In contrast, neither both short GST-tagged C termini, nor the three included negative controls (GST, GST-max p14, and GST-RIIβ), were able to pull-down HA-tagged RBP2. GAPDH served as another negative control as it was present in the cell lysates but was also not pulled down by any GST-tagged construct (Fig. 4b). The selective protein–protein interaction for GST-42_C-term_ with HA-RBP2 was observed despite partial protein degradation as visible in the Ponceau R staining (Fig. 4c, left panel). We did not observe interactions of HA-RBP2 with GST-42A_C-term_ (also showing partial degradation) or GST-43S_C-term_ (no degradation), suggesting no compromising influence on the detection of protein interactions by partial degradation of GST proteins. Using the anti-42 antibody (only recognizing the long C terminus, see Fig. 4a), we confirmed the presence of GST-42 on the same blot (Fig. 4c, right panel). Moreover, we also observed specific interaction of HA-tagged RBP2 with the long YFP-tagged C terminus (YFP-42) in co-immunoprecipitation experiments after co-expression of the interaction partners in tsA-201 cells (Fig. 4d). Taken together, we could confirm Cav1.3 splice variant-dependent RBP2 interaction with the distal C terminus, which is present only in the long Cav1.3 splice variant.

### Auxiliary β subunits in mouse cochlear IHCs

**Fig. 5:**
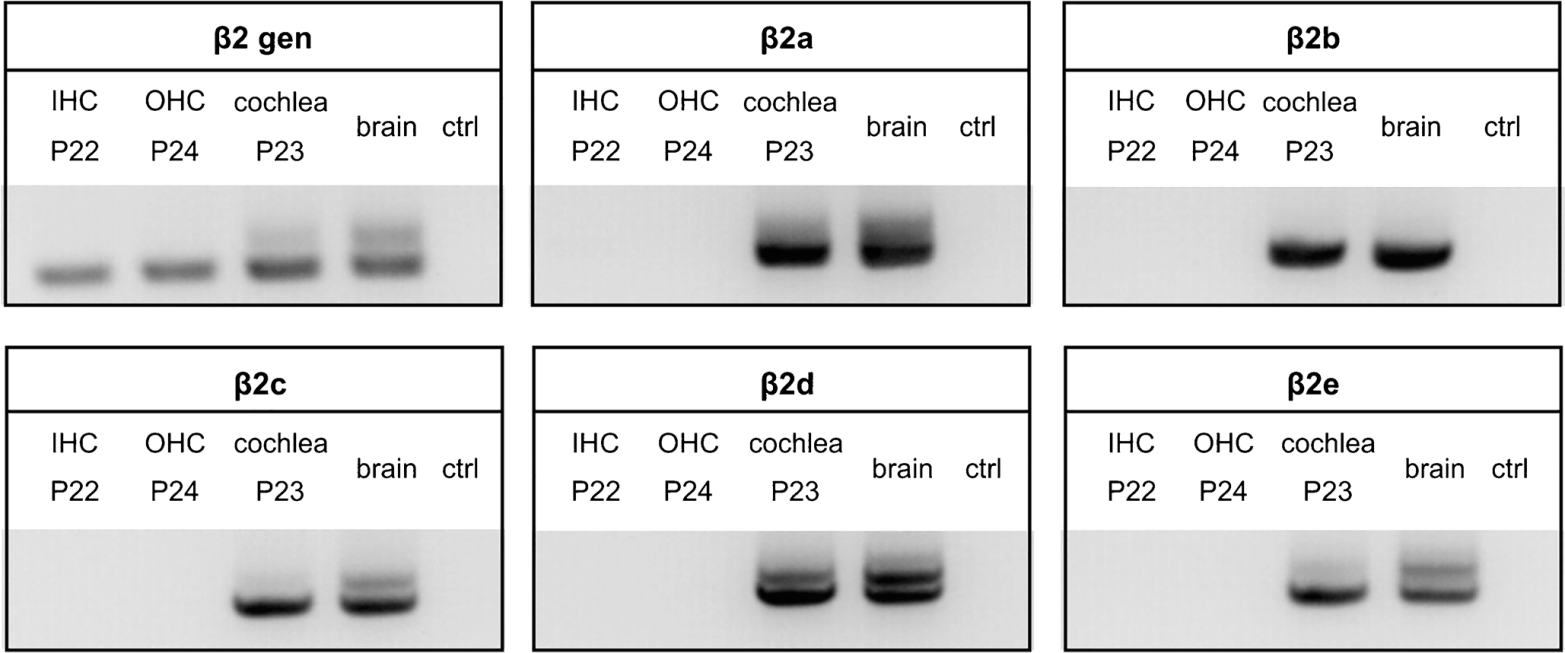
Detection of β2 subunit splice variants in mouse cochlea, IHC, and OHC preparations. β2 transcripts (β2gen) were detected in 4 of 8 independent experiments of 3 independent IHC samples. Its N-terminal splice variants were reproducibly detected in mouse cochlea preparations (8 of 8 independent experiments of at least 3 independent samples) and brain (4 of 4 independent experiments of at least 3 independent samples). However, the reproducible specific detection of N-terminal splice variants in IHC and OHC preparations using different primer combinations (see “Material and methods” section) was unsuccessful. Brain samples from adult mice and reactions without template (“ctrl”) were used as positive and negative controls, respectively

**Fig. 6:**
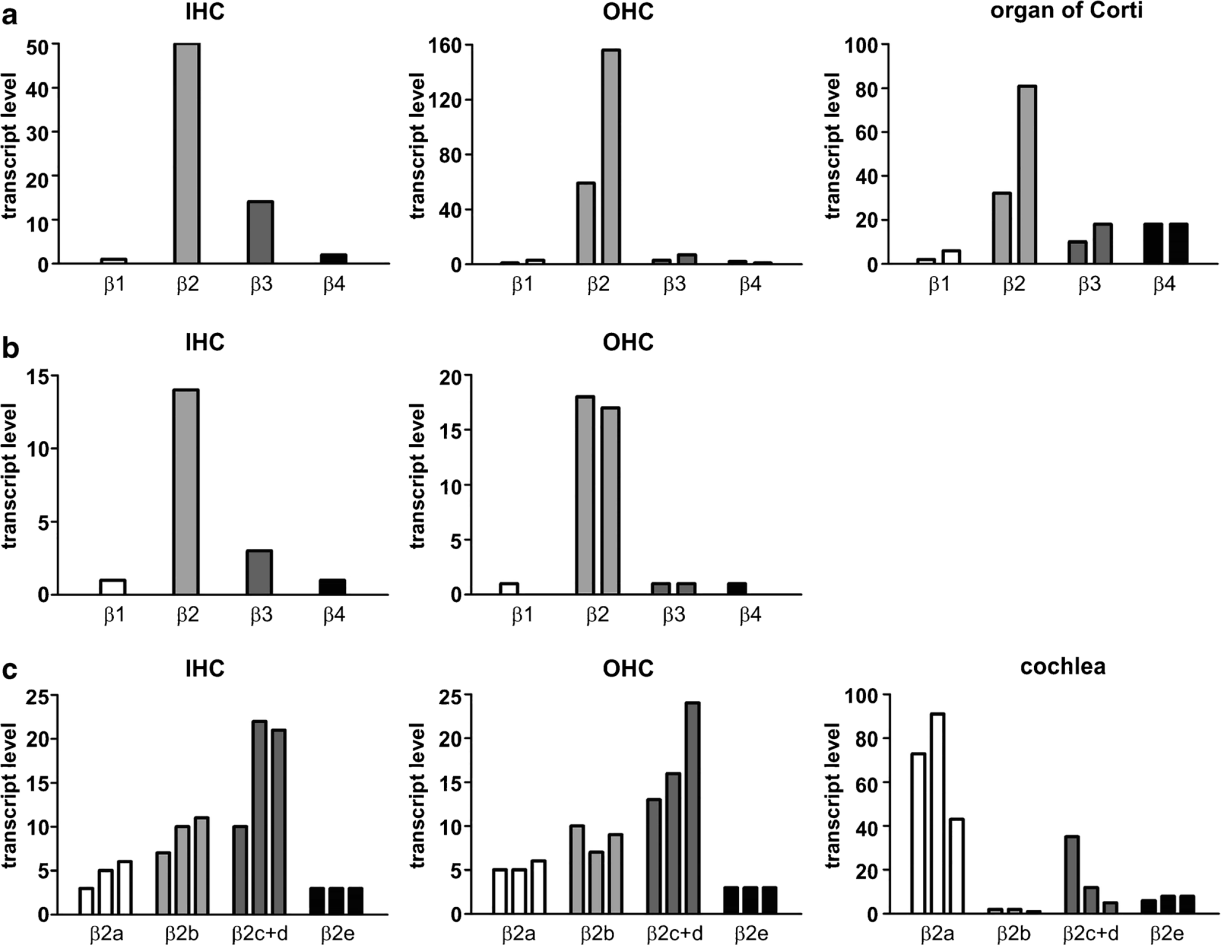
Expression levels of β1–4 isoforms and β2 splice variants. Results of individual experiments are illustrated. **a**, **b** Expression levels of β1–4 isoforms in IHCs, OHCs (**a** P6; **b** P20), and organ of Corti preparations (P5) from one IHC and two independent OHC and organ of Corti preparations. **c** Expression levels of N-terminal β2 splice variants in IHCs (P22), OHCs (P24), and whole cochlea (P23) preparations from three independent RNA preparations from three NMRI mice. IHC, inner hair cell; OHC, outer hair cell; P, postnatal

Having identified RBP2 and RIM2α as well as C-terminally long and short Cav1.3 α1 transcripts as potential interaction partners in IHCs, we investigated the expression profile of auxiliary LTCC β subunits. This is of particular importance since certain β subunit isoforms can also slow VDI [7] of VGCCs. However, quantitative PCR experiments of β subunit expression in IHCs have not been reported so far. Qualitative RT-PCR analysis [33, 42], evidence from β2-knockout mice [42], and a recent transcriptome analysis [35, 37] suggest that β2 subunits are the most abundant β subunit isoform in IHCs. Additionally, other isoforms, although expressed at lower levels [33, 35, 37, 42], must also support a substantial fraction of Cav1.3-mediated Ca^2+^ currents remaining in β2-deficient IHCs [42]. Among those, we and others reproducibly detected β3 subunits in IHC preparations [33, 42]. β3 subunits, like β1, β4, and the β2 N-terminal splice variants β2b, β2c, and β2d are not membrane-anchored through their N termini and therefore do not slow VDI [7, 16, 18, 27, 36]. However, reduced VDI has been reported for membrane-anchored β2a and β2e splice variants [7, 8, 16, 18, 27, 36]. Using qualitative PCR analysis, we could reliably identify all N-terminal β2 splice variants (β2a–β2e) in different mouse cochlea preparations (*n* = 3) and confirmed the presence of β2 subunits using a generic primer (β2gen) in both IHCs (9 out of 17 independent PCR reactions from 3 IHC preparations) and OHCs (4 out of 9 reactions, 2 preparations) (Fig. 5). OHCs, which also express Cav1.3 channels with very little VDI [29, 40], were used as an internal control. OHCs can be more easily obtained as individual cells with micropipettes due to the loose connection with their supporting Deiter’s cells, which easily breaks upon suction, and their 3-fold larger number compared with IHCs. However, we failed to reproducibly detect any of the β2 splice variants in hair cells despite using several independent hair cell preparations (*n* = 3) and primer pair combinations. This suggests that the assay sensitivity was not high enough to reliably detect individual splice variants in preparations of pooled isolated IHCs. Therefore, we established a standard curve-based quantitative real-time PCR (qRT-PCR) [54] using TaqMan gene expression assays (Table S2) in order to detect β1–β4 isoforms as well as β2a–β2e splice variants. As expected, β2 was the predominant isoform in hair cells (P6 and P20) and organ of Corti preparations (P5) in line with previous reports [42] (Fig. 6a, b). In addition, we identified β3 as well as β1 and β4 mRNA transcripts in pooled hair cells, which showed also low abundance in organ of Corti preparations. Next, we established custom TaqMan assays for β2 splice variants and were able to confirm for the first time the existence of “slow” β2a and β2e splice variants in IHCs and OHCs as well as in whole cochlea preparations. This result was highly reproducible in three independent RNA preparations of three biological replicates (Fig. 6c). In addition, we detected considerable amounts of β2b and β2c+d splice variants in all preparations. In summary, we could show for the first time that membrane-anchored “slow” β2 variants (β2a, β2e) account for ~ 15% of total β subunits in IHCs. For functional studies, we therefore employed β3 as a β subunit variant not slowing VDI, as well as β2a and β2e, known to stabilize slow VDI of Cav1.3.

### Modulation of Cav1.3 Ba^2+^ currents by RBP2

**Fig. 7:**
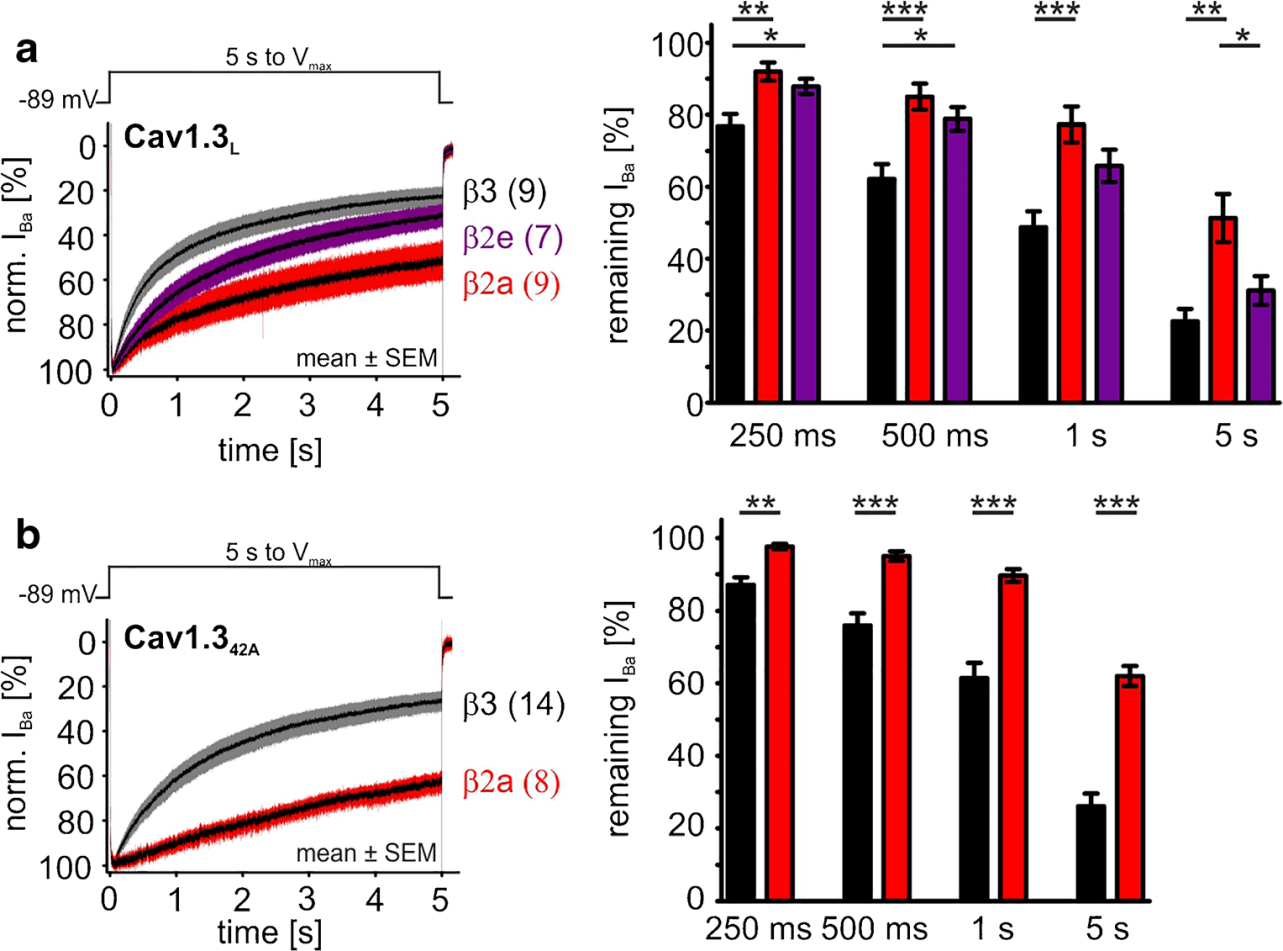
Modulation of VDI by β3 and different β2 subunit splice variants (15 mM Ba^2+^). **a**, **b** Left panels: mean (± SEM) *I*_Ba_ traces for Cav1.3_L_/α2δ1 (**a**) or Cav1.3_42A_/α2δ1 (**b**) co-expressed with either β3 (black/gray), β2a (red), or β2e (purple). The number of individual recordings is indicated in parentheses. VDI was quantified using 15 mM Ba^2+^ as charge carrier and calculated as residual *I*_Ba_ at the indicated predefined time points (bar graphs). Statistical significance was determined using one-way ANOVA with Bonferroni post hoc test (**a**) or unpaired Student’s *t* test (**b**): ***p < 0.001; **p < 0.01; *p < 0.05. For detailed statistics, see Table 2

Finally, we addressed the question of the functional consequences of the co-expression of functionally distinct β subunits as well as the here proposed VDI-slowing interaction partners RIM2α and RBP2. Therefore, we expressed human Cav1.3 α1 subunits with accessory α2δ1 and different β subunits together with RIM2α and/or RBP2 in tsA-201 cells and quantified the effects on gating in patch-clamp recordings. We used Ba^2+^ as the charge carrier to minimize CDI for quantification of VDI. Tagging RIM2α with GFP and RBP2 with RFP allowed identification of cells co-expressing one or both of these proteins. As expected, co-expression of β2a and β2e stabilized significantly slower *I*_Ba_ inactivation of Cav1.3_L_ (Fig. 7a) and Cav1.3_42A_ (Fig. 7b) compared to β3 as evident from the reduced inactivation during 5-s depolarizing pulses (left panels: mean current traces; right panels: statistics at predefined time points). Although the two β2 subunit splice variants both slowed VDI, β2a caused a strong inhibition over the whole time course of the 5-s long depolarization (Fig. 7a, b), whereas β2e only slowed the early time course of VDI but led to similar inactivation after 5 s as observed with β3 (shown for Cav1.3_L_ in Fig. 7a).

**Fig. 8:**
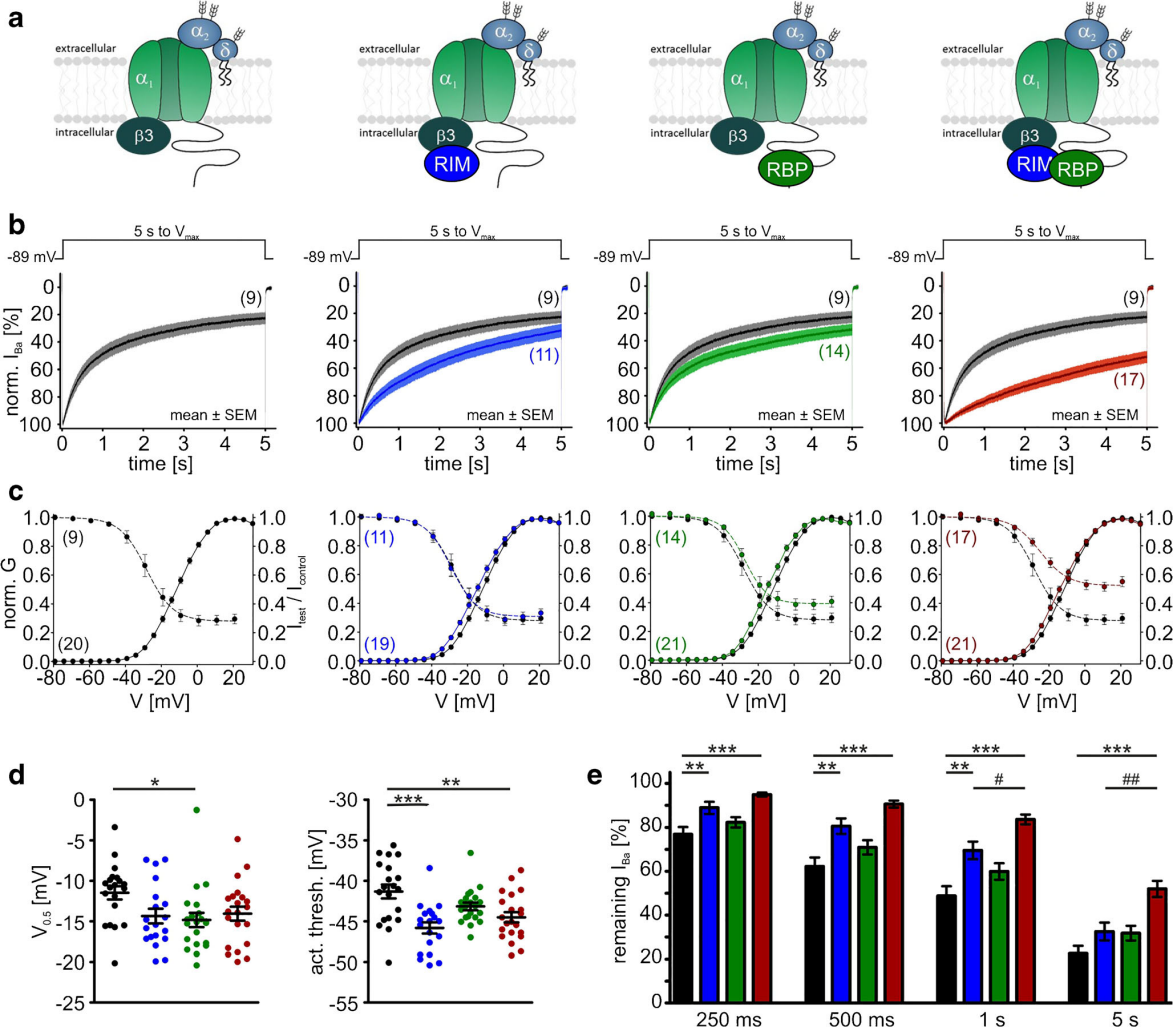
Modulation of Cav1.3_L_/α2δ1/β3 Ba^2+^ currents (15 mM) by co-expression of RIM2α and/or RBP2. **a** Schematic illustration of measured LTCC complexes, from left to right: control (Cav1.3_L_/α2δ1/β3); plus RIM2α; plus RBP2; plus RIM2α/RBP2. Data in panels **b** and **c** are shown for each recording condition. Color code: control (black), plus RIM2α (blue), plus RBP2 (green), and plus RIM2α/RBP2 (red). **b***I*_Ba_ inactivation time course during a 5-s long depolarization to the *V*_max_ (*y*-axis labels as in the left panel). Traces were normalized to the *I*_Ba_ peak and are shown as mean ± SEM for the indicated number of recordings. **c** Voltage dependence of *I*_Ba_ steady-state activation and inactivation (*y*-axis label as in the left panel). For parameters and statistics, see panel **d** and Table 1. **d** Statistics of two activation parameters (*V*_0.5,act_ and activation threshold) are shown. **e** Bar graphs showing the remaining *I*_Ba_ after 250, 500, 1000, or 5000 ms. Data shown as mean ± SEM. Statistical significance was determined using one-way ANOVA with Bonferroni’s multiple comparison post hoc test as indicated in the graph: versus control (Cav1.3 without RIM2α and/or RBP2): ***p < 0.001; **p < 0.01; *p < 0.05; RIM2α versus RIM2α/RBP2: ###*p* < 0.001; ##*p* < 0.01; #*p* < 0.05

**Fig. 9:**
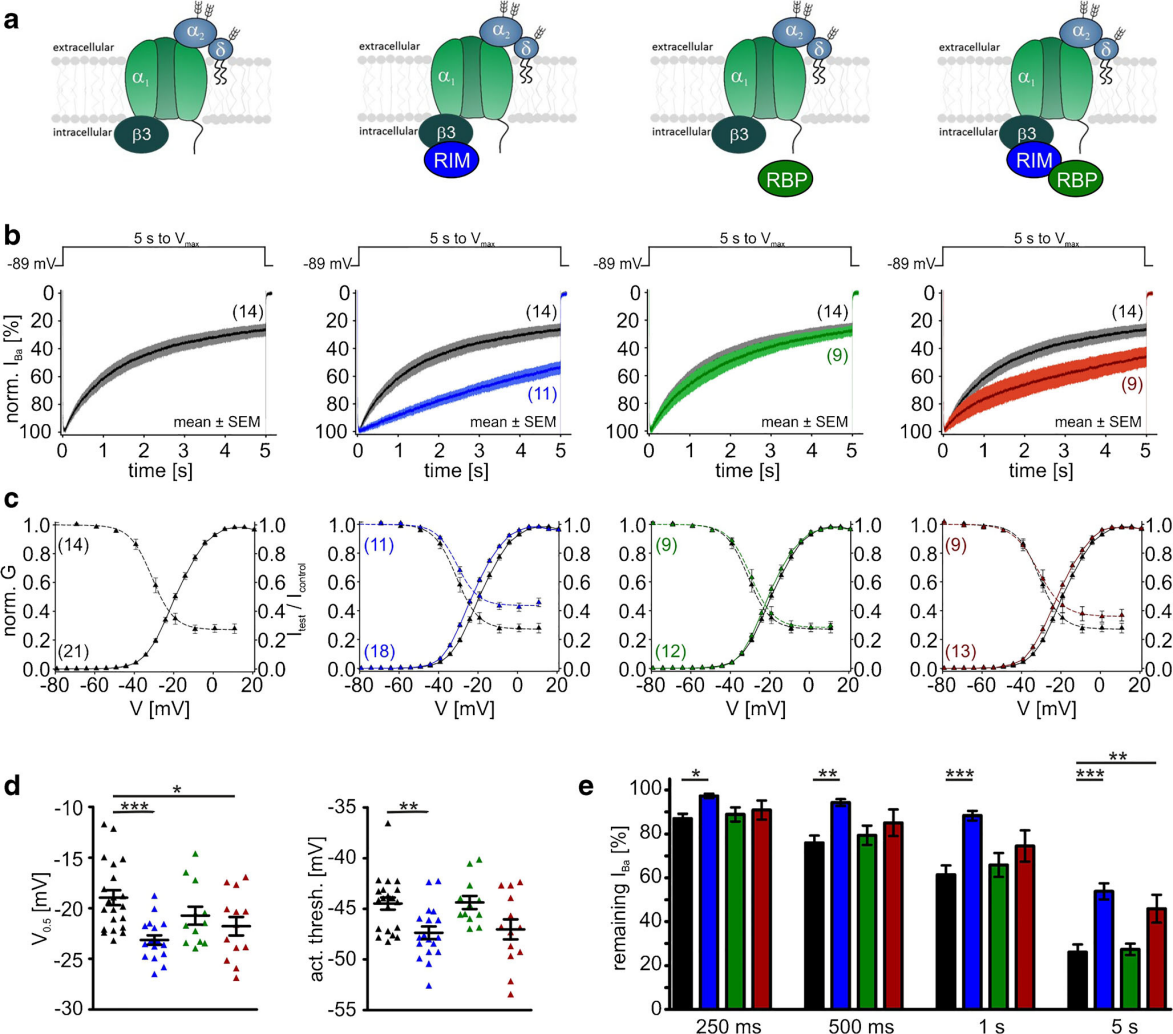
Modulation of Cav1.3_42A_/α2δ1/β3 Ba^2+^ currents (15 mM) by co-expression of RIM2α and/or RBP2. Color code: control (black), plus RIM2α (blue), plus RBP2 (green), and plus RIM2α/RBP2 (red). Experimental conditions and statistical analysis are as described in Fig. 8

In the presence of β3, RIM2α also significantly slowed the early time course of VDI of Cav1.3_L_ channels, but after 5 s, VDI was not significantly different from control (Fig. 8b, e for mean current traces and statistics at prespecified time points, respectively; Table 2). In contrast, RBP2 alone caused only a small slowing of VDI, without statistical significance at the prespecified time points (Fig. 8b, e). However, RBP2 co-expression together with RIM2α induced a strong slowing of VDI throughout the 5-s depolarization, which was significantly more pronounced than with RIM2α alone (Fig. 8b, e; Table 2) and resulted in more than 50% of remaining current after 5 s. Although RBP2 alone did not affect VDI, it reproducibly caused a significant 3–4-mV negative shift of the *V*_0.5,act_ indicating binding to the Cav1.3_L_ channel complex also in the absence of RIM2α (Fig. 8c, d for steady-state activation curves and statistics, respectively; Table 1). A similar significant shift of the activation threshold was also observed for RIM2α and RIM2α/RBP2 (Fig. 8c, d; Table 1). These data strongly support our hypothesis that simultaneous binding of RBP2 to both RIM2α and to the long Cav1.3 C terminus stabilizes slow IHC-like VDI. The crucial role of the Cav1.3 C terminus for modulation by RIM2α and RBP2 was evident from co-expression with Cav1.3_42A_, which lacks the RBP2 interaction domain (Fig. 1 and Fig. 4). When co-expressed with Cav1.3_42A_, RIM2α alone already caused a marked inhibition of VDI, indistinguishable from RIM2α/RBP2 co-expressed with Cav1.3_L_ (Fig. 9b, e; Table 2). It also shifted the steady-state activation (*V*_0.5,act_ and activation threshold) by about 3–4 mV to more negative voltages (Fig. 9c, d; Table 1). As expected from the absence of the RBP2-binding motif in the short Cav1.3_42A_ C terminus, RBP2 alone had no effect on VDI (Fig. 9b, e; Table 2) or the channel’s voltage dependence of activation (Fig. 9c, d; Table 1). In contrast to Cav1.3_L_, RBP2 did not enhance, but even tended to reduce RIM2α-induced slowing of VDI upon co-expression (Fig. 9b, e; Table 2).

**Table 1:** Parameters of the voltage dependence of steady-state activation (left) or inactivation (right) of Cav1.3_L_ or Cav1.3_42A_ LTCCs measured with α2δ1 and different β subunits (β3, β2a, β2e) in the presence or absence of RIM2α and/or RBP2

Construct	Activation				Inactivation			
	*V*_0.5,act_ [mV]	*k*	Act thresh [mV]	*n*	*V*_0.5,inact_ [mV]	*k*_inact_	Plateau [%]	*n*_inact_
Cav1.3_L_/β3	− 11.5 ± 0.8	7.8 ± 0.1	− 41.3 ± 0.9	20	− 27.6 ± 2.2	6.0 ± 0.3	27.9 ± 3.4	9
+ RIM2α	− 14.4 ± 0.9	8.1 ± 0.1	− 45.8 ± 0.7***	19	− 28.4 ± 1.7	6.3 ± 0.5	30.6 ± 2.8	11
+ RBP2	− 14.8 ± 0.9*	7.6 ± 0.2	− 43.2 ± 0.5	21	− 25.6 ± 2.0	5.5 ± 0.2	38.1 ± 3.5	14
+ RIM2α/RBP2	− 14.1 ± 0.9	8.1 ± 0.2	− 44.5 ± 0.6**	21	− 23.5 ± 1.3	7.6 ± 0.5	50.8 ± 3.1***^,###^	17
Cav1.3_42A_/β3	− 19.0 ± 0.7	6.9 ± 0.2	− 44.5 ± 0.6	21	− 30.6 ± 1.3	4.8 ± 0.2	27.4 ± 2.9	14
+ RIM2α	− 23.1 ± 0.5***	6.6 ± 0.1	− 47.4 ± 0.6**	18	− 30.6 ± 0.6	5.1 ± 0.3	44.1 ± 2.8***	11
+ RBP2	− 20.7 ± 0.9	6.5 ± 0.2	− 44.4 ± 0.6	12	− 29.2 ± 1.4	5.1 ± 0.5	28.6 ± 2.7	9
+ RIM2α/RBP2	− 21.8 ± 0.9*	6.8 ± 0.1	− 47.0 ± 1.0	13	− 30.9 ± 1.6	5.6 ± 0.5	36.4 ± 3.9	9
Cav1.3_L_/β2a	− 7.2 ± 1.4	9.4 ± 0.1	− 44.1 ± 1.1	17	− 16.5 ± 2.3	13.0 ± 0.8	47.6 ± 5.6	9
+ RIM2α	− 9.2 ± 1.8	9.1 ± 0.2	− 44.4 ± 1.4	9	− 17.3 ± 4.3	11.3 ± 1.2	45.1 ± 4.6	7
+ RBP2	− 11.0 ± 1.2	9.1 ± 0.2	− 46.2 ± 1.3	10	− 18.8 ± 1.9	11.1 ± 0.7	51.8 ± 2.5	6
+ RIM2α/RBP2	− 12.2 ± 1.0*	9.2 ± 0.2	− 48.0 ± 0.8*	17	− 20.0 ± 2.2	10.9 ± 0.5	52.4 ± 3.6	10
Cav1.3_L_/β2e	− 7.5 ± 2.1	8.8 ± 0.3	− 41.9 ± 1.2	11	− 24.0 ± 2.8	7.5 ± 0.8	25.6 ± 4.3	7
+ RIM2α	− 13.5 ± 1.5*	8.9 ± 0.2	− 47.8 ± 1.0**	11	− 22.4 ± 1.6	9.0 ± 0.4	42.4 ± 3.3*	8
+ RBP2	− 7.8 ± 0.8	9.1 ± 0.2	− 43.0 ± 1.0	11	− 21.1 ± 2.0	9.7 ± 1.0	32.2 ± 7.2	6
+ RIM2α/RBP2	− 15.6 ± 1.4**	8.5 ± 0.1	− 47.7 ± 1.0**	15	− 24.6 ± 2.5	8.5 ± 0.7	42.6 ± 2.5*	10
Cav1.3_42A_/β2a	− 22.2 ± 1.2	7.5 ± 0.2	− 50.2 ± 1.1	14	− 32.4 ± 1.8	6.7 ± 0.6	60.9 ± 3.5	8
+ RIM2α	− 23.2 ± 0.6	7.3 ± 0.3	− 50.2 ± 1.4	14	− 35.0 ± 1.4	5.4 ± 0.3	49.1 ± 4.3	10
+ RBP2	− 21.9 ± 1.1	7.0 ± 0.1	− 47.5 ± 1.3	13	− 32.2 ± 1.7	5.8 ± 0.5	56.0 ± 4.3	11
+ RIM2α/RBP2	− 23.3 ± 0.8	7.3 ± 0.2	− 50.5 ± 1.1	16	− 34.0 ± 1.6	6.5 ± 0.9	45.8 ± 3.0*	10

Data are given as means ± SEM. Statistical significance was determined by one-way ANOVA with Bonferroni’s multiple comparison post hoc test as indicated in the table. Versus control (Cav1.3 without RIM2α and/or RBP2): ***p < 0.001; **p < 0.01; *p < 0.05; RIM2α versus RIM2α/RBP2: ###*p* < 0.001

*V*_*0.5,act*_, voltage of half-maximal activation; *k*, slope factor; *act thresh*, activation threshold (voltage where 5% of maximal *I*_Ba_ is reached); *V*_*0.5,inact*_, voltage of half-maximal inactivation; *k*_*inact*_, inactivation slope factor

**Table 2:** Voltage-dependent inactivation (VDI) during a 5-s depolarizing pulse from a HP of − 89 mV to *V*_max_ was quantified by calculating the residual Ba^2+^ current fraction after 250, 1000, or 5000 ms (r250, r1000, r5000)

Construct	r250 [%]	r500 [%]	r1000 [%]	r5000 [%]	*n*
Cav1.3_L_/β3	76.9 ± 3.3	62.2 ± 4.1	48.8 ± 4.4	22.6 ± 3.5	9
+ RIM2α	88.9 ± 2.7**	80.6 ± 3.5**	69.6 ± 4.0**	32.6 ± 4.1	11
+ RBP2	82.4 ± 2.4	70.9 ± 3.3	59.9 ± 3.8	31.8 ± 3.3	14
+ RIM2α/RBP2	94.9 ± 0.9***	90.6 ± 1.6***	83.7 ± 2.3***^,#^	52.0 ± 3.7***^,##^	17
Cav1.3_42A_/β3	87.1 ± 2.1	76.0 ± 3.3	61.4 ± 4.2	26.1 ± 3.5	14
+ RIM2α	97.3 ± 1.0*	94.3 ± 1.6**	88.4 ± 2.1***	53.8 ± 3.6***	11
+ RBP2	88.9 ± 3.1	79.4 ± 4.4	65.9 ± 5.5	27.3 ± 2.6	9
+ RIM2α/RBP2	90.9 ± 4.3	85.1 ± 6.0	74.6 ± 7.3	45.9 ± 6.4**	9
Cav1.3_L_/β2a	92.0 ± 2.6	85.0 ± 3.6	77.3 ± 5.0	51.3 ± 6.7	9
+ RIM2α	91.1 ± 1.9	85.1 ± 3.2	77.7 ± 4.4	48.6 ± 6.6	7
+ RBP2	92.3 ± 0.4	85.7 ± 0.8	77.8 ± 0.9	49.5 ± 1.9	6
+ RIM2α/RBP2	92.6 ± 1.0	86.4 ± 1.7	79.3 ± 2.5	52.2 ± 3.9	10
Cav1.3_L_/β2e	87.9 ± 2.1	78.9 ± 3.3	65.9 ± 4.5	31.1 ± 4.0	7
+ RIM2α	90.8 ± 1.8	84.0 ± 2.5	70.0 ± 2.9	40.4 ± 3.4	8
+ RBP2	85.3 ± 4.7	77.3 ± 6.9	64.5 ± 9.1	34.8 ± 8.5	6
+ RIM2α/RBP2	87.9 ± 2.3	81.4 ± 3.1	73.0 ± 3.7	45.4 ± 3.4	10
Cav1.3_42A_/β2a	97.6 ± 0.8	95.0 ± 1.3	89.6 ± 1.8	62.0 ± 2.8	8
+ RIM2α	96.5 ± 1.0	92.3 ± 1.6	85.6 ± 2.6	49.9 ± 3.8	10
+ RBP2	97.6 ± 0.9	94.6 ± 1.5	89.1 ± 2.2	59.9 ± 3.7	11
+ RIM2α/RBP2	96.7 ± 1.0	92.8 ± 1.5	85.8 ± 2.0	52.3 ± 2.8	10

Data are given as means ± SEM. Statistical significance was determined by one-way ANOVA with Bonferroni’s multiple comparison post hoc test as indicated in the table. Versus control (Cav1.3 without RIM2α and/or RBP2): ***p < 0.001; **p < 0.01; *p < 0.05; RIM2α versus RIM2α/RBP2 ##*p* < 0.01; #*p* < 0.05

**Fig. 10:**
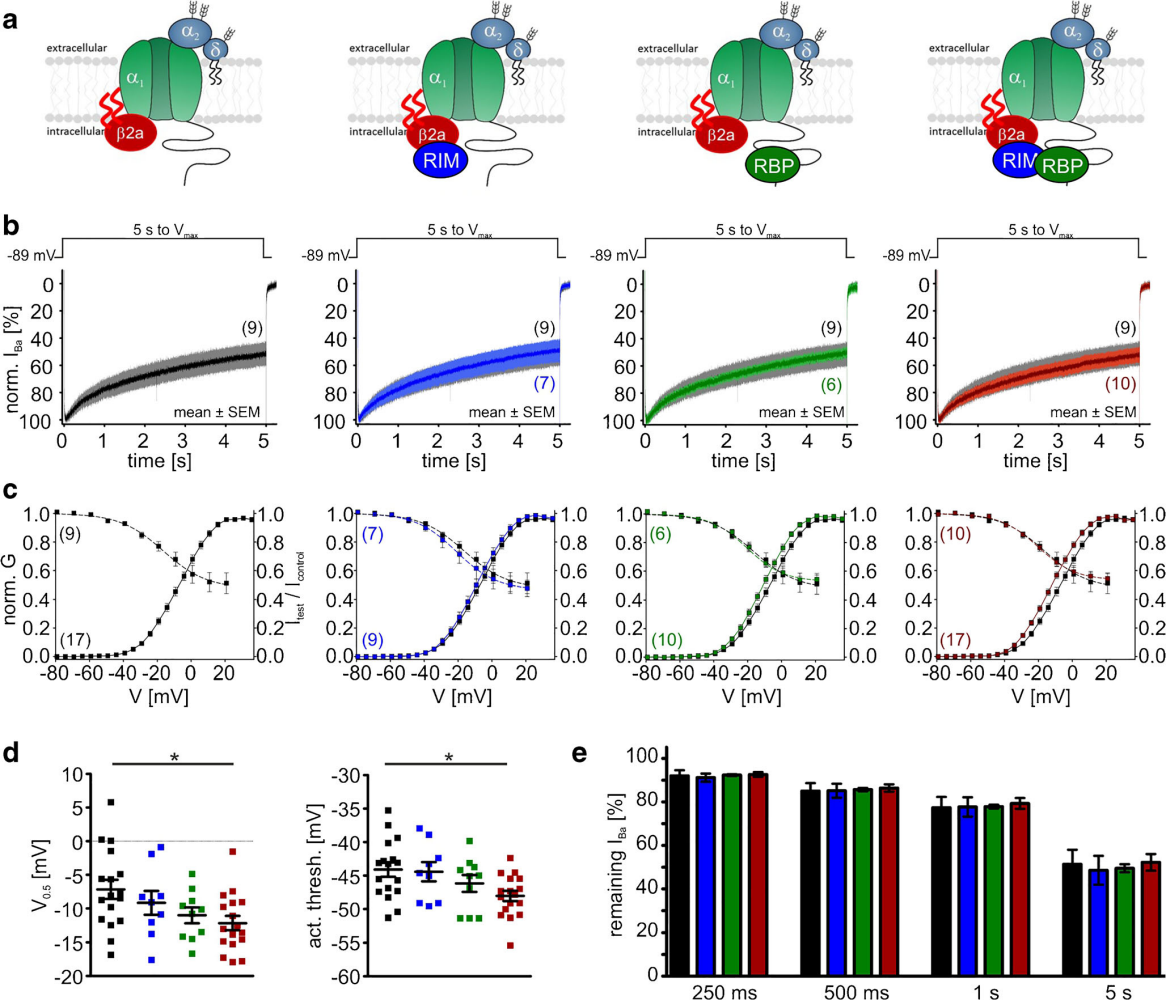
Modulation of Cav1.3_L_/α2δ1/β2a Ba^2+^ currents (15 mM) by co-expression of RIM2α and/or RBP2. Color code: control (black), plus RIM2α (blue), plus RBP2 (green), and plus RIM2α/RBP2 (red). Experimental conditions and statistical analysis are as described in Fig. 8

**Fig. 11:**
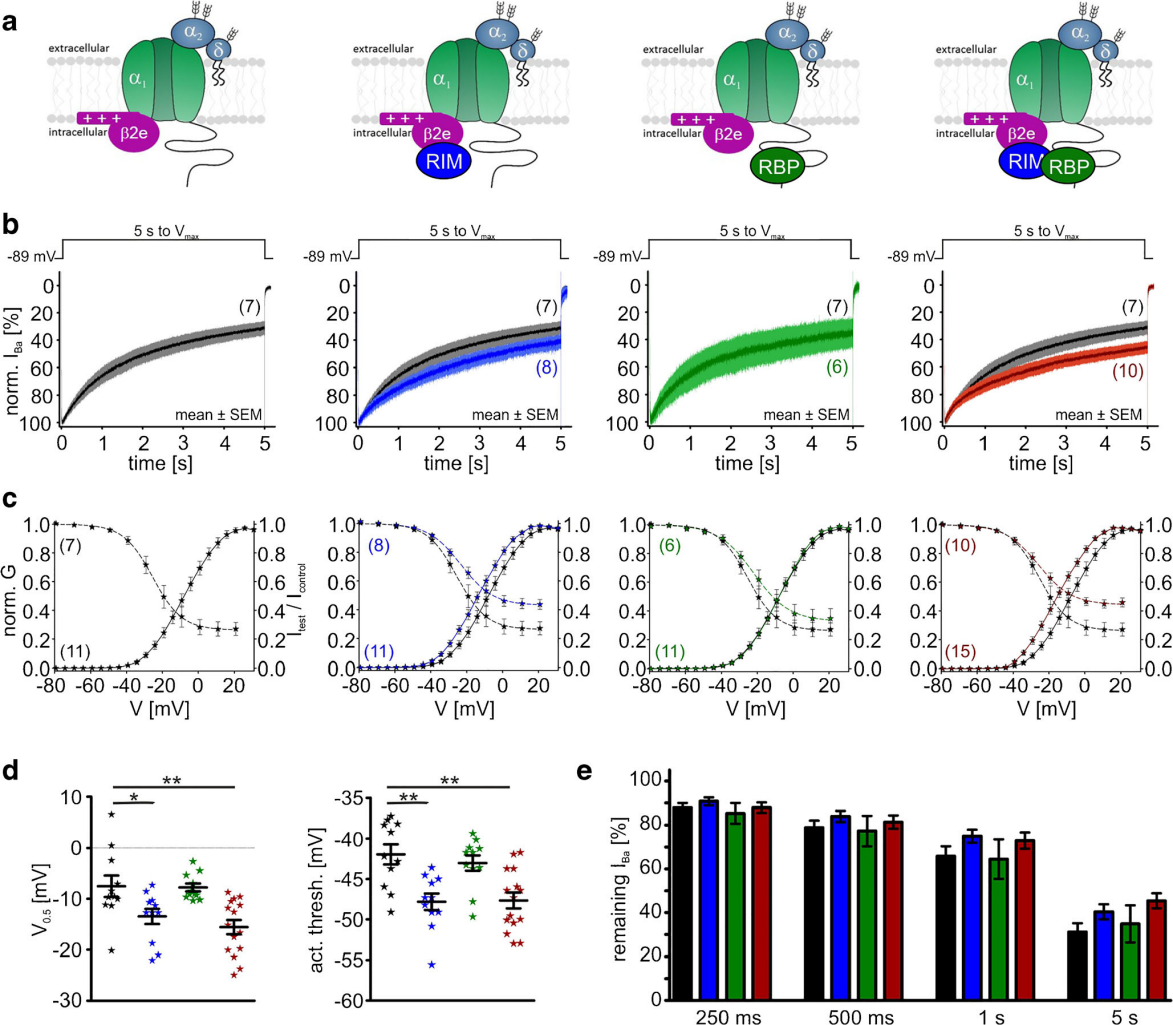
Modulation of Cav1.3_L_/α2δ1/β2e Ba^2+^ currents (15 mM) by co-expression of RIM2α and/or RBP2. Color code: control (black), plus RIM2α (blue), plus RBP2 (green), and plus RIM2α/RBP2 (red). Experimental conditions and statistical analysis are as described in Fig. 8

**Fig. 12:**
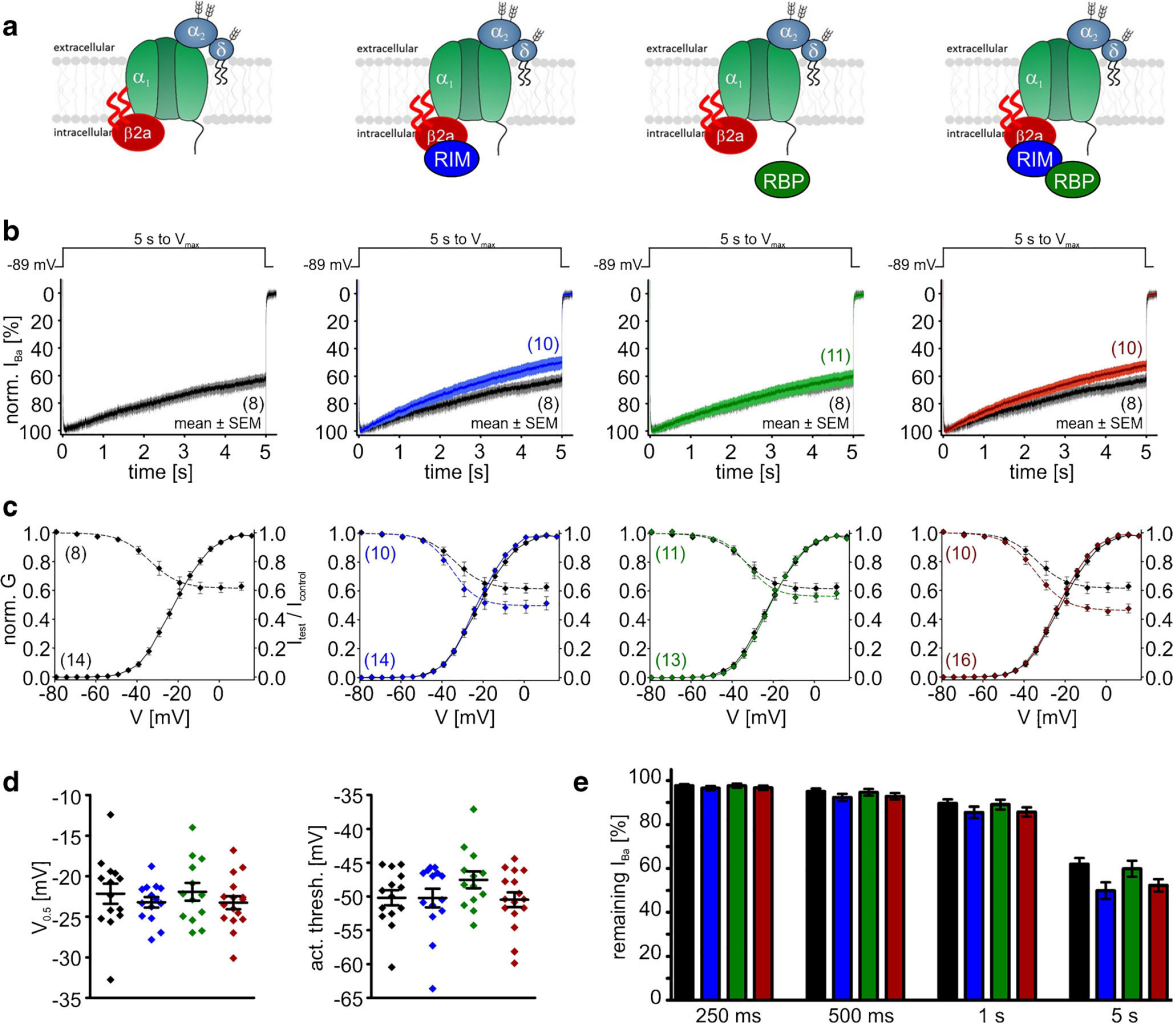
Modulation of Cav1.3_42A_/α2δ1/β2a Ba^2+^ currents (15 mM) by co-expression of RIM2α and/or RBP2. Color code: control (black), plus RIM2α (blue), plus RBP2 (green), and plus RIM2α/RBP2 (red). Experimental conditions and statistical analysis are as described in Fig. 8

So far, we have found a VDI-slowing effect by co-expression of RIM2α (Cav1.3_42A_) or RIM2α/RBP2 (Cav1.3_L_) when β3 was part of the channel complex. This was indistinguishable from Cav1.3_L_ and Cav1.3_42A_*I*_Ba_ inactivation kinetics when co-expressed with the “slow” β2a subunit alone (Table 2). Therefore, we next studied if VDI of β2a or β2e-containing Cav1.3 channel complexes was further modulated by RIM2α, RBP2, or both. In contrast to β3, the slow VDI of the long Cav1.3 splice variant associated with β2a was not further modulated by RIM2α (Fig. 10b, e; Table 2). This is in agreement with earlier findings by us and others [18, 49]. However, also no further slowing was induced by RBP2 (Fig. 10b, e). For β2e, only a weak slowing by RIM2α (without and with RBP2) was observed (Fig. 11b, e), which was also evident as a significant increase of the noninactivating current component (“plateau”) in steady-state inactivation curves (Fig. 11c, Table 2 for statistics). Again, Cav1.3 C-terminal splicing affected modulation: For β2a-stabilized Cav1.3_42A_ channels, RIM2α and RIM2α/RBP2 tended to slightly accelerate VDI (Fig. 12b, e; Table 2) and RIM2α/RBP2 shifted the voltage dependence of activation (*V*_0.5,act_ and activation threshold) of β2a-containing Cav1.3_L_, but not of Cav1.3_42A_ (Table 1 for statistics).

Our data also show that C-terminal splicing affects the regulation of Cav1.3 channels by different β subunits. Long Cav1.3 C-terminal splice variants activate at more positive voltages than short variants (Table 1) [4, 44, 57, 62]. This is mainly due to a weaker voltage sensitivity of activation evident as a larger slope factor for activation and, as described here, was also observed when β2a is part of the channel complex (Table 1). However, we found that β2a subunits have opposite effects on the *V*_0.5,act_ of Cav1.3_L_ and Cav1.3_42A_. As compared to β3, β2a shifted *V*_0.5,act_ of Cav1.3_L_ to more positive voltages (β3 − 11.5 mV; β2a − 7.2 mV; *p* = 0.017; Table 1). This difference disappeared in the presence of RIM2α/RBP2. In Cav1.3_42A_, however, β2a slightly shifted *V*_0.5,act_ to more negative voltages (β3 − 19.0 mV; β2a − 22.2 mV; *p* < 0.0001, Table 1), an effect that was not prevented by RIM2α/RBP2.

**Fig. 13:**
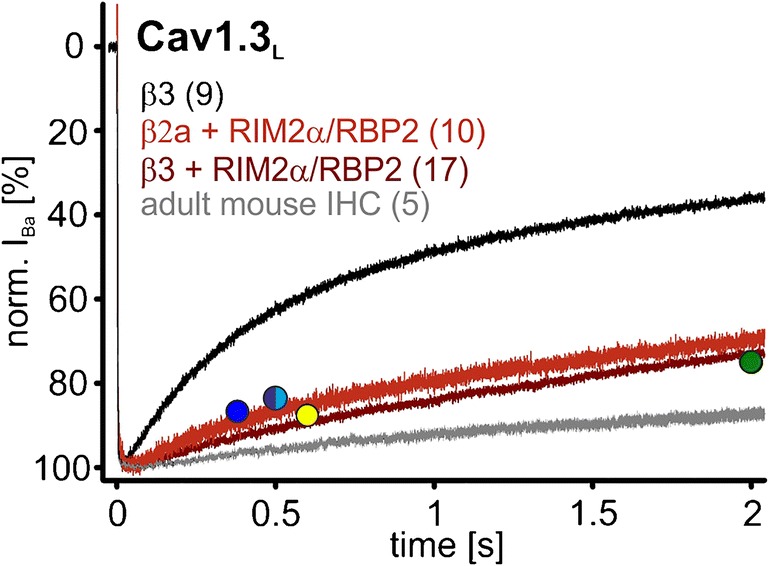
Comparison of RIM2α/RBP2-stabilized Cav1.3_L_*I*_Ba_ inactivation (β3 and β2a; tsA-201 cells) with *I*_Ba_ VDI measured in IHCs. Mean *I*_Ba_ (15 mM) traces of Cav1.3_L_/α2δ1 with β3 (black), β3/RIM2α/RBP2 (dark red), or β2a/RIM2α/RBP2 (red) during the first 2 s of a depolarization to *V*_max_. For comparison, we recorded *I*_Ba_ in mature mouse IHCs measured as recently described [53] in mature mouse IHCs (mean *I*_Ba_ trace from 5 individual recordings; gray; 10 mM Ba^2+^). Circles indicate the remaining Ba^2+^ current at the indicated time points recorded from IHCs taken from previously published papers: [33] (dark blue; 10 mM Ba^2+^, mouse P20); [42] (turquoise; 5 mM Ba^2+^, mouse P40–70); [9] (purple; 5 mM Ba^2+^, mouse 2–4 weeks); [24] (yellow; 5 mM Ba^2+^, gerbil P50); [34] (green; 20 mM Ba^2+^, chicken 1–21 days). Turquoise and purple circles are overlapping and are therefore shown together as half-filled circle

Taken together, our data strongly suggest that presynaptic scaffolding proteins together with accessory β subunits stabilize the unique slow VDI, which is the limiting step for current inactivation in cochlear IHCs. As illustrated in Fig. 12, the inactivation time course of Cav1.3_L_/α2δ1/β3 and of Cav1.3_L_/α2δ1/β2a channels in complex with RIM2α/RBP2 is well within the range of inactivation kinetics of Ba^2+^ currents recorded in adult mouse IHCs (10 mM Ba^2+^, *n* = 5, Fig. 13) and as previously reported in IHCs by different laboratories (Fig. 13). Moreover, despite a common effect on VDI, different β subunit isoforms in combination with Cav1.3 C-terminal splicing and RIM2α and/or RBP2 modulate the voltage dependence of Cav1.3 activation over a wide voltage range. This likely contributes to the variability of voltage dependence of presynaptic Ca^2+^ influx at individual ribbon synapses observed in IHCs [43].

## Discussion

Here we provide strong evidence that the association of Cav1.3 channels with RIM2α/RBP2 and/or with membrane-anchored β subunit splice variants can account for the slow inactivation kinetics that are a prerequisite for the proper function of cochlear IHCs during sound-induced tonic neurotransmitter release. Effects of RIM2α alone [18, 26] and of β2a subunits on Cav1.3 channel inactivation [18, 31, 42] have been described before. We now show that RBP2 plays an additional crucial role for stabilizing two essential properties of native Cav1.3-mediated currents in IHCs: slow VDI as well as the negative voltage activation range.

VDI is the limiting process for inactivation in IHCs because CDI is almost completely suppressed by CaM-like Ca^2+^-binding properties [67] of proteins such as CaBP2 [55]. In tsA-201 cells, we show that the effects of RIM2α/RBP2 on inactivation strongly depend on the extent of VDI already stabilized by the associated β subunit. Because β3 subunits support fast VDI, the cooperative action of RIM2α/RBP2 allows inhibition of VDI. In contrast, β2a itself, through its palmitoylation membrane anchor, strongly inhibits VDI and thus prevents further modulation not only by RIM2α but also by RIM2α/RBP2. These mechanisms together ensure slow VDI—independent of Cav1.3 C-terminal splicing and of associated β subunits—resulting in a similar fraction (45–52%) of current remaining at the end of 5-s depolarizations when RIM2α and RBP2 are part of the channel complex (Table 2).

Differences in the inactivation kinetics of “fast” (such as β3) or “slow” (such as β2a) β subunits could either be achieved by an increase of intrinsically slow Cav1.3 VDI by fast β subunits, or by a reduction of intrinsically fast VDI by slow β subunits. Preliminary experiments indicate that Cav1.3 LTCCs possess an intrinsically slow inactivation in the absence of an auxiliary β subunit. This indicates that β3 subunits increase VDI and RIM2α/RBP2 or β2a prevents the β3 effect. To prove this hypothesis, it would require a more extensive analysis of β-free LTCC complexes, which is, however, complicated by the fact that current densities are very small in the absence of β.

Moreover, we show that while stabilizing slow VDI, the different β subunit isoforms together with RIM2α and RBP2 are able to fine-tune the voltage dependence of Cav1.3 activation over a wide voltage range. RIM2α/RBP2 can shift activation gating to more negative voltages, in particular of C-terminally long Cav1.3_L_ channels, which activate at more positive voltages than Cav1.3_42A_ [44, 62]. This is interesting in a developmental context, because immature IHCs did not yet express RBP2 (Fig. 2). A shift of the activation threshold of RIM2α/RBP2-coupled presynaptic Cav1.3_L_ channels toward more negative voltages would increase the sensitivity to low sound pressure levels. The most sensitive response to sound is accomplished by ribbon synapses coupled to type Ia spiral ganglion neurons [47, 56, 60]. Because these neurons have a high spontaneous rate and are easily saturated by moderate sound pressure levels, very slow VDI as well as CDI are necessary for their indefatigable function in hearing mice. It is important to note that our experiments in transiently transfected tsA-201 cells required recordings with 15 mM Ba^2+^ as the charge carrier, which shifts the voltage dependence of gating by about 5–10 mV to more positive potentials as compared to physiological Ca^2+^ concentrations ([66, 71]; NJ. Ortner, unpublished observations). This puts the foot of the activation curve of Cav1.3 well within the IHC operation voltage range of approximately − 60 to − 35 mV [13, 19]. Therefore, even small negative shifts, as reported here, will facilitate channel activation in particular at low sound pressure levels inducing small depolarizations of the receptor potential. We also show that the stabilization of more positive half-activation voltages of Cav1.3 by C-terminal splicing (higher *V*_0.5,act_ of Cav1.3_L_ vs Cav1.3_42A_) and also by its association with β2a (vs β3) subunits occurs predominantly by decreasing the voltage sensitivity of gating, as evident by larger slope factors (less steep activation curves). Instead, the shift of activation gating to more negative voltages by RIM2α/RBP2 occurs mainly by lowering activation thresholds while keeping the voltage sensitivity unchanged.

The combination of C-terminal splicing and different β subunits gives rise to slowly inactivating RIM2α/RBP2-associated channels all activating within a narrow range of activation thresholds (between − 44.5 and − 50.5 mV, Table 1) but with very different voltage sensitivities (e.g., Cav1.3_42A_/β3 vs Cav1.3_L_/β2a). In IHCs, this heterogeneity may also contribute to the pronounced variation of the voltage dependence of Cav1.3-dependent presynaptic Ca^2+^ influx at individual active zones of IHCs [43]. Each IHC needs to code sound intensity over a wide dynamic range (80 dB), which appears to rely on the functional differentiation of the ~ 15 release sites each driving a spiral ganglion neuron [43]. Indeed, a differentiation of spiral ganglion neurons type I into subtypes Ia, Ib, and Ic, which are characterized by high, medium, or low spontaneous rate and are thought to code for soft, medium, or loud sounds, respectively, has been identified recently [47, 56, 60]. One possible explanation for the functional variation of ribbon synapses of the same IHCs is the differences in the voltage dependence of active zone Cav1.3 Ca^2+^ channels, which are major determinants of both spontaneous and sound-driven firing of spiral ganglion neurons [13, 43], but other presynaptic as well as postsynaptic differences may also be [47, 60] involved. Although our data cannot explain the large range of activation thresholds for presynaptic Ca^2+^ influx observed at individual active zones, they can explain the large differences in the voltage sensitivity of Ca^2+^ influx observed at a given activation threshold [43].

Direct proof for our hypothesis would come from biochemical studies demonstrating the presence of different Cav1.3 α1 and β subunit splice variants together with RIM2α and RBP2 in individual active zones. However, such experiments are currently impossible due to the absence of suitable splice variant-specific antibodies for β2 subunits and antibodies specific for short Cav1.3 splice variants. We have previously reported a first attempt to study the splice variant-specific expression of Cav1.3 in IHCs by generating a mutant mouse expressing a hemagglutinin–antibody tag only within the long C-terminal tail [53]. This allowed localization of Cav1.3_L_ at all CtBP2/ribeye-positive synaptic ribbons. However, these experiments do not exclude the concomitant presence of different levels of short Cav1.3 splice variants. As evident from a recently published transcriptome analysis [35, 37], expression levels of Cav1.3 α1 (Cacna1d) and β2 (Cacnb2) transcripts are about 20–100-fold lower than of other IHC proteins, such as harmonin (Ush1c), otoferlin (otof), or Kv1.8 K^+^ channels (Kcna10). However, by applying a standard curve-based qRT-PCR approach, we detected all N-terminal β2 splice isoforms in cochlear IHCs (Fig. 6) and could thereby prove for the first time the presence of “slow” β2a and β2e subunits. As shown here, in the presence of RIM2α and RBP2, slow VDI is also stabilized when “slow” β subunits (β2a, β2e) are absent and only “fast” β subunits (all other isoforms) that also account for a substantial fraction of hair cell β subunits are present. This is also in excellent agreement with the observation that despite an about 70% decrease of IHC Ca^2+^ current amplitude in β2-subunit-deficient mice, the inactivation time course remains slow [42].

Taken together, in IHCs, a broad repertoire of mechanisms ensures the unique slow Cav1.3 VDI, which is a prerequisite for tonic sound-induced neurotransmitter release—including the association with RIM2α/RBP2 and/or “slow” β subunits. Yet, our data do not exclude a role for other active zone proteins in stabilizing slow Cav1.3 channel inactivation. RBP2 knockout mice showed only a mild auditory deficit and slightly reduced IHC Ca^2+^ currents with unchanged activation gating and Ca^2+^ current inactivation kinetics [32]. However, RBP2 deficiency might be compensated by other RBP isoforms, such as RBP3 (which we show to be expressed at all developmental stages), or other presynaptic active zone proteins. Although RBP3 has been described as testis-specific protein [41, 72] and does not show the typical upregulation during postnatal IHC (Fig. 2) or brain development [41] like other synaptic proteins, a potential compensatory role cannot be excluded. An inducible hair cell-specific RBP2 knockout model may therefore help to specifically study the function of RBP2 for hearing. The scaffold protein harmonin can interact with Cav1.3 by binding to the PDZ-binding domain of the long C terminus of Cav1.3_L_, but has not been shown to be important for slow inactivation in IHCs [43]. Unfortunately, we could not study an additional role of CaM-like Ca^2+^ binding proteins, in particular CaBP2, which appears to be the predominant isoform inhibiting CDI in IHCs [35, 37, 55]. The additional co-expression of CaBP2 with all accessory Cav1.3 subunits together with RIM2α and RBP2 would be experimentally very challenging and, in addition, complicated due to cytotoxic properties of this protein [67].

## Electronic supplementary material

ESM 1 (DOCX 55.7 kb)
