# Supplementary material for: RBP2 stabilizes slow Cav1.3 Ca2+ channel inactivation properties of cochlear inner hair cells
Source: Pflugers Arch. 2019 Dec 17;472(1):3–25. doi: 10.1007/s00424-019-02338-4 (PMC6960213; doi:10.1007/s00424-019-02338-4)
Supplement: Supplementary file 1 — (DOCX 55.7 kb) [file 424_2019_2338_MOESM1_ESM.docx]

Supplementary material

**Pflügers Archiv – European Journal of Physiology**

RBP2 stabilizes slow Cav1.3 Ca^2+^ channel inactivation properties of cochlear inner hair cells

**Nadine J. Ortner^1*^, Alexandra Pinggera^1^, Nadja T. Hofer^1^, Anita Siller^1^, Niels Brandt^2^, Andrea Raffeiner^3^, Kristina Vilusic^1^, Isabelle Lang^2^, Kerstin Blum^2^, Gerald J. Obermair^4,5^, Eduard Stefan^3^, Jutta Engel^2^, and Jörg Striessnig^1*^**

^1^ Department of Pharmacology and Toxicology, Institute of Pharmacy, Center for Molecular Biosciences Innsbruck, University of Innsbruck, Innsbruck, Austria

^2^ Department of Biophysics and CIPMM, Saarland University, Homburg, Germany

^3^ Institute of Biochemistry, Center for Molecular Biosciences Innsbruck, University of Innsbruck, Innsbruck, Austria

^4^ Division of Physiology, Medical University Innsbruck, Innsbruck, Austria

^5^ Karl Landsteiner University of Health Sciences, Division Physiology, Krems, Austria

*** Correspondence:**

Nadine J. Ortner ([nadine.ortner@uibk.ac.at](mailto:nadine.ortner@uibk.ac.at); +43-512-507-58815) or Jörg Striessnig ([joerg.striessnig@uibk.ac.at](mailto:joerg.striessnig@uibk.ac.at); +43-512-507-58800)

| **Outer primer** | **Sequence 5´-3´** | **Fragment size** | **Binding site** | **Genbank accession #** |
| --- | --- | --- | --- | --- |
| β2gen out fwd-1 | TCAGATGTGTCTTTGGAAGAG | 319 | Exon 3 NT | XM_011238946.2 |
| β2gen out fwd-2 | CTTGCCGACTATCTGGAGGCATACTG |  |  |  |
| β2a out fwd | ATGCAGTGCTGCGGGCTGGT | 403 | Exon 2A NT | XM_011238946.2 |
| β2b out fwd | TGCTTGACAGGCAGTTGGTG | 405 | Exon 2C NT | NM_001252533.1 |
| β2c out fwd | TGGACCAGGCGAGTGGACTG | 483 | Exon 1B NT | XM_006497314.3 |
| β2d out fwd | ATGGTCCAAAGCGACACGTC | 556 | Exon 1A NT | NM_023116.4 |
| β2e out fwd | ATGAAGGCCACCTGGATCAG | 424 | Exon 2D NT | NM_001309519.1 |
| β2gen out rev-1 | CTCTCTGTTCGTGCTGTAGC |  | Exon 5 Hook |  |
| β2gen out rev-2 | CAGCTACCCAAACAGCTTTTGAATTGTTCAGG |  |  |  |
| β2gen out rev-3 | CTCTAGTTTGACTGGGCTTGGA |  |  |  |
| RIM1α fwd | CCGACGTGTGGAATCTGTC | 464 |  | NM_053270 |
| RIM1α rev | CTTGGTGGCTCACTTCTTGAC |  |  |  |
| RIM2α fwd | TGCAGCAACCTGATCAAAAG | 572 |  | NM_053271 |
| RIM2α rev | TGCAGCAACCTGATCAAAAG |  |  |  |
| RBP1 fwd | AAATGCCCGCCTGGTGGAGG | 752 |  | NM_172449.2 |
| RBP1 rev | TGGCTGTGTCCACCTCTGGTGT |  |  |  |
| RBP2 fwd | CTGAGCTGCCTCTCACGGCG | 678 |  | NM_001081388.1 |
| RBP2 rev | TGTTGGTCGGCAGCCACGAG |  |  |  |
| RBP3 fwd | CAGAAGCCTGTTGGCTGGGCT | 768 |  | NM_001033338.3 |
| RBP3 rev | CAGGGCCACGAGGACTGGACT |  |  |  |
| Cav1.3 fwd | GGGCCAGAAATCCGACGGGC | 624 (43L)  470 (43S) |  | NM_001083616 |
| Cav1.3 rev | TCCAGGTGGGAGAGCTGTCGT |  |  |  |
| **Inner primer** | **Sequence 5´-3´** | **Fragment size** | **Binding site** | **Genbank accession #** |
| β2gen in fwd-1 | AGTACGTAGAGAAGCTGAGC | 224 | Exon 3 NT | XM_011238946.2 |
| β2gen in fwd-2 | CACCCTCCCAGCGGTAACC |  |  |  |
| β2a in fwd | GAGTACGGGTGTCCTATGGT | 309 | Exon 2D NT | XM_011238946.2 |
| β2b in fwd | GACTCAATCCAGTATTCCTGG | 314 | Exon 2D NT | NM_001252533.1 |
| β2c in fwd | AGATCTCCTATGGAAAAGGAGC | 391 | Exon 1B-2A NT | XM_006497314.3 |
| β2d in fwd | CAGATGGAGCTGCTGGAGAG | 436 | Exon 1A NT | NM_023116.4 |
| β2e in fwd | GAGGAAGGCTGAAGAGTTCG | 321 | Exon 2D NT | NM_001309519.1 |
| β2gen in rev-1 | CACAGCCTTCTTTAACCAGC |  | Exon 5 SH3 |  |
| β2gen in rev-2 | GCTATTGGAGCAACAGCAATACAAAATAGAC |  |  |  |
| β2gen in rev-3 | CGTCCTATCCACCAGTCATTAT |  |  |  |
| RIM1α fwd | GGCCATCTCTGCTCCTATTG | 387 |  | NM_053270 |
| RIM1α rev | CTTCTGTTCAGGACCCAAGG |  |  |  |
| RIM2α fwd | AGCCCCTCAGGAGAAGAAAG | 477 |  | NM_053271 |
| RIM2α rev | TAGCGTGCCTGGTATTCCTC |  |  |  |
| RBP1 fwd | GCATATGCGGGAGGTGGCCC | 406 |  | NM_172449.2 |
| RBP1 rev | GGAGCCCGGTGGGAGGTCAA |  |  |  |
| RBP2 fwd | GTCGCGGCTGGCTGGTACTC | 410 |  | NM_001081388.1 |
| RBP2 rev | TCGTGACGCACTGCACGGAG |  |  |  |
| RBP3 fwd | TGTCACGGCTACCTCCGCCA | 416 |  | NM_001033338.3 |
| RBP3 rev | TGGCGTCGGCAACTTCGGTC |  |  |  |
| Cav1.3 fwd | ACGAGCCAGAAGACTCCAAA | 557 (43L)  403 (43S) |  | NM_001083616 |
| Cav1.3 rev | CACAGCACTCCTCGCTACTG |  |  |  |

**Table S1:** cDNA specific primers for nested PCR: gen, generic primer to detect all β2 variants. The primers β2gen out fwd-2, β2gen out rev-2, β2gen in fwd-2 and β2gen in rev-2 were as in [41]. NT, N-terminus. Fragment sizes are given for forward primers in combination with their respective reverse primers (for β2gen: given for reverse primer β2gen out rev-1).

| **Subunit**  **variant** | **Genbank**  **accession**  **number** | **Assay ID or sequences**  **for custom assay** | **Exon**  **boundary** | **Primer sequences for standard template cloning/amplification** | **Fragment**  **length (bp)** |
| --- | --- | --- | --- | --- | --- |
| Cavβ1 | NM_031173.4 | Mm00518940_m1 | 2/3 | Fwd: 5´- GATCCTCTCCATGGTCCAGAA-3´  Rev: 5´-CTGCCTCCTTCCTTAAGGCTTC-3´ | 266 |
| Cavβ2 | NM_023116.4 | Mm00659092_m1 | 13/14 | Fwd: 5´- GACTATCTGGAGGCATACTGGAAG-3  Rev: 5'-CTCTCTTGGGTTTCAGAGTCAAA-3’ | 317 |
| Cavβ3 | NM_007581.3 | Mm00432233_m1 | 1/2 | Fwd: 5´- CCCATGTATGACGACTCCTACG-3  Rev: 5'-ACAGTAGCTGACATTGGTCCTCAC-3’ | 216 |
| Cavβ4 | NM_146123.3 | Mm00521623_m1 | 11/12 | Fwd: 5´- GCTGATTAAGTCCAGAGGAAAGTC-3  Rev: 5'-TGTCTCATTCGCTGACTCTGTAAT-3’ | 288 |
| Cavβ2a | XM_011238946.2 | Fwd: 5´-CACGGTGCCGCTTGGT-3´  Rev: 5´-TGCATGAAGAGGTGGCAGAA-3´  Probe: 5´-AAGCCACGCTCTGAC-3´ | 5´-UTR-ex2B | Fwd: 5´-ATATGTCGACTGGACAGTGGCCGTGACGAG-3  Rev: 5´-CTTGAAGCTTCACAGCCTTCTTTAACCAGC-3´* | 440 |
| Cavβ2b | NM_001252533.1 | Fwd: 5´-TTACACATCTCAAACTTCAGGGAAAA-3´  Rev: 5´-CCAGCTAAAGGTGGCTTTGC-3´  Probe: 5´-CGGAGCCCGTGCGA-3´ | 5´-UTR | Fwd: 5´-ATATGTCGACCGTTAGAAAGTCATGAAGTC-3´  Rev: 5´-CTTGAAGCTTCACAGCCTTCTTTAACCAGC-3´* | 523 |
| Cavβ2c+d | XM_006497314.3  NM_023116.4 | Fwd: 5´-AAAGGCTCGGATGGAAGCA-3´  Rev: 5´-CCCTGGCGGACAAAACTGT-3´  Probe: 5´-ATCGTCAGACACTACCTC-3´ | ex2A-3 | Fwd: 5´-ATATGTCGACTTGCCGATGGTCCAAAGCGAC-3´  Rev: 5´-CTTGAAGCTTCACAGCCTTCTTTAACCAGC-3´* | 519 |
| Cavβ2e | NM_001309519.1 | Fwd: 5´-GGGAGGAAGGCTGAAGAGTTC-3´  Rev: 5´-GGGCGGCTGGTGTAGGA-3´  Probe: 5´-ACATCTGTGGTTCGGC-3´ | ex2D-3 | Fwd: 5´-ATATGTCGACCTACCCGGCTCATGAAGGCCACCTGG-3´  Rev: 5´-CTTGAAGCTTCACAGCCTTCTTTAACCAGC-3´* | 392 |
| **Table S2**: TaqMan assays for Cavβ1-4 isoforms and N-terminal β2 splice variants and cDNA specific primer sequences for standard template amplification/cloning. Additional *Sal*I and *Hin*dIII restriction enzyme (RE) sites were introduced (underlined); * the reverse (rev) primer with *Hin*dIII RE site was the same for β2a-e fragments; fwd, forward. | | | | | |

| **Subunit variant** | **B** | **SE-B** | **Y-int.** | **SE-Y** | **R^2^** | **LOD** | **LOQ** | **E (%)** |
| --- | --- | --- | --- | --- | --- | --- | --- | --- |
| Cavβ1 | -3.474 | 0.06 | 36.34 | 0.04 | 0.996 | 3 | 34 | 94 |
| Cavβ2 | -3.491 | 0.04 | 39.48 | 0.03 | 0.990 | 6 | 190 | 93 |
| Cavβ3 | -3.519 | 0.13 | 37.58 | 0.20 | 0.993 | 12 | 114 | 92 |
| Cavβ4 | -3.535 | 0.25 | 38.01 | 0.18 | 0.992 | 5 | 137 | 92 |
| Cavβ2a | -3.283 | 0.037 | 35.04 | 0.54 | 0.981 | 10 | 100 | 101 |
| Cavβ2b | -3.334 | 0.070 | 34.36 | 0.14 | 0.990 | 10 | 120 | 99 |
| Cavβ2c+d | -3.160 | 0.023 | 35.78 | 0.02 | 0.998 | 5 | 20 | 107 |
| Cavβ2e | -3.070 | 0.005 | 35.30 | 0.12 | 0.993 | 5 | 50 | 111 |

**Table S3**: Standard curve parameters. SE-B,-Y, standard error of B and Y-intercept; Y-int., Y-intercept (C_T_ value); R^2^, squared correlation coefficient; LOD, limit of detection (number of transcripts); LOQ, limit of quantification (number of transcripts); E (%), Efficiency in % (E=10^-1/slope^-1), 100% efficiency corresponds to a slope of -3.32

| **Primer** | **Forward primer**  **Sequence 5´-3´** | **Reverse primer**  **Sequence 3´-5´** |  |
| --- | --- | --- | --- |
|  |  |  | |
| HA-rRBP2 PCRa | TGCCCACTTGGCAGTACATC | AGCGTAATCTGGAACATCGTATGGGTACATGGCGACCGGTAGCGCTAGCG | |
| HA-rRBP2 PCRb | ATGTACCCATACGATGTTCCGATTACGCTCTCAAGCTTATGCGAGAGGC | TGGATGGAAGTGGCTAGTCC | |
| HA-rRBP2 PCRc | TGCCCACTTGGCAGTACATC | TGGATGGAAGTGGCTAGTCC | |
|  |  |  | |
| GST-Cav1.3 42_C-term_ | GTCGTGGGATCCCCGATAATTTCGACTATCTGACCCGGGACTGGTCTATTTTGGGGCCTC | GATGCGGCCGCTCGAGCTACAAGGTGGTGATGCATATCATTTCATCC | |
|  |  |  | |
| GST-Cav1.3 42A_C-term_ | GTCGTGGGATCCCCGATAATTTCGACTATCTGACCCGGGACTGGTCTATTTTGG | GATGCGGCCGCTCGAGCTAGAGCATCCGTTCAAGCATCTGTAGGGCGATCGTGG | |
|  |  |  | |
| GST-Cav1.3 43S_C-term_ | GTCGTGGGATCCCCGATAATTTCGACTATCTGACCCGGGACTGGTCTATTTTGG | GATGCGGCCGCTCGAGCTATGGAATTATGGTTATGATGGTTATGACACACC | |

**Table S4:** Specific forward and reverse primers for cloning of rRBP2 and GST-Cav1.3 cDNA constructs are given.
